# Animals devoid of pulmonary system as infection models in the study of lung bacterial pathogens

**DOI:** 10.3389/fmicb.2015.00038

**Published:** 2015-02-04

**Authors:** Yamilé López Hernández, Daniel Yero, Juan M. Pinos-Rodríguez, Isidre Gibert

**Affiliations:** ^1^Centro de Biociencias, Universidad Autónoma de San Luis PotosíSan Luis de Potosí, Mexico; ^2^Institut de Biotecnologia i de Biomedicina, Universitat Autònoma de BarcelonaBarcelona, Spain; ^3^Departament de Genètica i de Microbiologia, Universitat Autònoma de BarcelonaBarcelona, Spain

**Keywords:** alternative model, pneumonia, zebrafish, *C. elegans*, *Drosophila melanogaster*, *Galleria mellonella*, tuberculosis, cystic fibrosis

## Abstract

Biological disease models can be difficult and costly to develop and use on a routine basis. Particularly, *in vivo* lung infection models performed to study lung pathologies use to be laborious, demand a great time and commonly are associated with ethical issues. When infections in experimental animals are used, they need to be refined, defined, and validated for their intended purpose. Therefore, alternative and easy to handle models of experimental infections are still needed to test the virulence of bacterial lung pathogens. Because non-mammalian models have less ethical and cost constraints as a subjects for experimentation, in some cases would be appropriated to include these models as valuable tools to explore host–pathogen interactions. Numerous scientific data have been argued to the more extensive use of several kinds of alternative models, such as, the vertebrate zebrafish (*Danio rerio*), and non-vertebrate insects and nematodes (e.g., *Caenorhabditis elegans*) in the study of diverse infectious agents that affect humans. Here, we review the use of these vertebrate and non-vertebrate models in the study of bacterial agents, which are considered the principal causes of lung injury. Curiously none of these animals have a respiratory system as in air-breathing vertebrates, where respiration takes place in lungs. Despite this fact, with the present review we sought to provide elements in favor of the use of these alternative animal models of infection to reveal the molecular signatures of host–pathogen interactions.

## INTRODUCTION: A GOOD ANIMAL MODEL

To study the pathology, host immune response and the complex interactions between host and pathogen, the use of animal models have been invaluable, but for many years, it have presented strong public, scientific concerns, as well as philosophical contradictions (Lipscomb et al., 2010). From the great contributions of Luis Pasteur and Robert Koch in the use of animal models to decipher the causal agents of several diseases, including *Bacillus anthracis*, *Mycobacterium tuberculosis* or the rabies virus, concepts related to the animal use and handle and bioethics have arise (Baumans, 2004). Russell and Burch (1959) developed the “Three Rs” principle (Replacement, Reduction, and Refinement), although their work remained largely ignored well into the 1970s (Balls and Halder, 2002). At this time, rodents were considered despicable animals and consequently they were treated without consideration, wide spreading its use as research model (Damy et al., 2010). In 1999, the Declaration of Bologna reaffirmed that “humane science is a prerequisite for good science, and is best achieved in relation to laboratory animal procedures by the vigorous promotion and application of the Three Rs” (Balls, 2009). In 2002, the genome sequence of mouse was completed (first mammal after humans; Watkins-Chow and Pavan, 2008). This fact largely contributed to the great use of mouse as animal model. However, and taken into account the principle of Replacement, several alternative models far different from the mammals classic ones, have been developed in the last years (Hendriksen, 2002).

A perfect animal model would be a model that satisfied not only scientific, but also ethical criteria (Zak and O’Reilly, 1993). Among the most controversial experimental animal models from the point of view of ethics, lung infections induced by bacteria are considered by far the most aggressive. For instance, several pathogens are able to kill non-human mammals due to lung infections. For this reason, these models need to be optimized to better reproduce human infections and acute lung damages. In addition, when an animal model is used in preclinical research, we have to consider that not all results will be successfully extrapolated from animal studies to humans (Fuchs et al., 2009; Evans et al., 2010). Some important anatomical, physiological, genetic, and molecular differences are clearly present between species.

The pathogenesis of infection is a result of the balance of the host–pathogen interaction (Mason et al., 1995). The pathogen and host genetic backgrounds are a relevant determinant of this outcome, as continuously several genes are activated or repressed depending on environmental changes during experiments. For many years it was thought that mammalian models of infection were the unique choice to study host–pathogen interactions as well as for pre-clinical evaluation of vaccines and drugs before their use in humans (Means and Aballay, 2011). However, there are presently numerous scientific data that have been argued to the more extensive use of several kinds of alternative infection models, such as, small vertebrates, insects, and nematodes (Kurz and Ewbank, 2007). The present review summarizes, compare and discuss the published experience in classical animal models, such as mice, and alternative animal models, particularly the zebrafish (*Danio rerio*), *Caenorhabditis elegans* and the insects *Drosophila melanogaster* and *Galleria mellonella* (the greater wax moth) in the study of bacterial agents which are considered the principal causes of lung injury.

## ANIMAL MODELS TO STUDY BACTERIAL LUNG PATHOGENS, SAFETY AND ETHICAL ISSUES AND THE NEED OF ALTERNATIVE METHODS

### OVERVIEW OF MAMMALIAN MODELS FOR RESEARCH ON PULMONARY INFECTION

The respiratory system of most non-human mammals mimics in general a lung environment in humans, in terms of chemical and physical conditions and spatial structures. In addition, the pulmonary defenses to respiratory infections in non-human mammals are somehow similar to humans. Hence, most of the pneumonia animal studies are carry out in mammals to facilitate some types of investigation (Mizgerd and Skerrett, 2008). However, extrapolation of results to humans is not straightforward owing to significant anatomical and physiological differences between species. The different mammals do not appear to present similar mucociliary clearance and alveolar macrophage morphometry (Fernandes and Vanbever, 2009).

To produce experimental pulmonary infection, mammalians offer a wide diversity of inoculation routes [reviewed and compared in Bakker-Woudenberg (2003)], being intranasal (i.n.) and intratracheal (i.t.) inoculations the infection routes seem to be the most “naturally acquired.” The intratracheal model of infection requires a complex and invasive technique for disease induction but offers the advantage of allowing almost total delivery of the bacterial inoculum to the lungs. The model of infection through intranasal aspiration is the most commonly used, as it is fast and easier to perform without invasive surgical procedures and also because it mimics the natural route of infection in humans. When the inoculum is administered intranasally it could be applied as an aerosol (passive inhalation) or as nasal droplets. The intranasal aerosol model instead requires an exposure chamber with a nebulizer, and permits the simultaneous infection of several mice (Mizgerd and Skerrett, 2008). However, aerosol studies carry the greatest potential risk of infection with airborne pathogens (Chen et al., 2011), the inhaled dose varies considerably and the equipment to perform the infection is not always available in research laboratories. Most of these routes may require anesthetize animals and sometimes post-administration of pain relief drugs.

Rodents, more than other animals, are commonly used to study pneumonia. Mice are the most used in infection experiments and offer many advantages, including that they are relatively inexpensive, easy to maintain, easy to handle and their genome can be manipulate (Denny, 2005; Leung et al., 2013). Despite of this, surgical and invasive techniques are required to reproduce some acute or chronic lung diseases in mice. However, the use of general anesthesia with their side effects in host is considered as a great disadvantage of these techniques (Mizgerd and Skerrett, 2008). Guinea pigs, rats and hamsters apart of mice, are some of the other animals employed as models of lung infections (Bakker-Woudenberg, 2003).

There are well-documented relevant differences relative to lung infection between mice and humans. Differences in the structural anatomy, cellular composition of the tracheobronchial epithelium, local phagocytic and chemical defenses, inflammatory or immune response, need to be analyzed at the moment of interpreting the experimental results. Mice are different in terms of their lower complexity of the airway branches and a relatively large caliber of the airway lumen (Irvin and Bates, 2003). Furthermore, they have different cellular expression and ligand binding for selected Toll like receptors (TLRs; Mizgerd and Skerrett, 2008; Apt and Kramnik, 2009). In contrast with humans, this species has a well-developed broncus associated lymphoid tissues (BALTs) system, may be due to their life habits. In healthy humans, this system is practically absent (Hyde et al., 2009). However, the availability of immunological reactives as well as several mice transgenic lines has made their use almost indispensable in the majority of infection studies.

Mice are resistant (not susceptible) too many human pathogens (Lyons et al., 2004; Di Pietrantonio and Schurr, 2005; Aziz et al., 2007). Consequently, in addition to rodents, several other mammalian animal models have been explored in respiratory diseases studies: cattles, goats, sheeps, pigs, dogs, and non-human primates. The fact that non-human mammalian species are more phylogenetically related to humans justifies at least in part the use of these species by researchers. However, none of them completely reproduces all the aspects of lung diseases in humans. The most clinically relevant model has been the primate infection models, due to the high genetic and structural similarity with humans (Bem et al., 2011; Kaushal et al., 2012). Nonetheless, the cost, time, logistic and ethical considerations and the risk of zoonoses of this model mean that it can only be used in a limited fashion.

Despite the simplicity of their immune system, and their evolutionary distance to human, some non-mammalian models using small animals (fishes, nematodes, and insects) are characterized by their short generation time which redundance in the low cost of experiments. Curiously none of these animals have a respiratory system as in air-breathing vertebrates, where respiration takes place in lungs. However, such models have allowed successful screening for virulence genes in the most common bacterial lung pathogens (**Table 1**).

**Table 1:** Most significant contributions of alternative animal models for the study of some relevant lung pathogens.

Lung pathogen	Alternative model	Relevant contribution to pulmonary infection in mammals	Reference
*Streptococcus pneumoniae*	Zebrafish embryos	Pneumococci evade immune clearance by interfering with phagocytic functions.	Rounioja et al. (2012)
	Adult zebrafish	Virulence attenuated mutants are defective in polysaccharide capsule, autolysin, or pneumolysin.	Saralahti et al. (2014)
	*Galleria mellonella* larvae	Non-capsulated and pneumolysin defective strains were less virulent than their respective wild types. The role of antimicrobial peptide activity and resistance has been addressed.	Evans and Rozen (2012)
*Staphylococcus aureus*	Zebrafish embryos	Role of Macrophages in the ingestion of *S. aureus* during *in vivo* infections.	Prajsnar et al. (2008)
	*Caenorhabditis elegans*	Several genes encoding virulence factors, including biofilm-related, identified in *S. aureus* were relevant for mammalian pathogenesis.	Sifri et al. (2003, 2006), Begun et al. (2005, 2007)
	*G. mellonella* larvae	Both bacterial glycolysis and gluconeogenesis have important roles in virulence.	Purves et al. (2010)
*Mycobacterium tuberculosis*	Zebrafish infected with *Mycobacterium marinum*	Infection with *M. marinum* induces host proteases in epithelial cells and enhances recruitment of macrophages in order to promote granuloma formation to facilitate bacterial dissemination.	Volkman et al. (2010)
	Zebrafish infected with *M. marinum*	The importance of Th2-type response in controlling mycobacterial infection.	Hammarén et al. (2014)
	*Drosophila melanogaster* infected with *M. marinum*	Model to reveal the relationship between phagocytes and bacteria.	Dionne et al. (2003)
*Pseudomonas aeruginosa*	*C. elegans* (Slow killing)	Positive correlation between *P. aeruginosa* genes required to kill *C. elegans* and those for pathogenesis in mammals.	Tan et al. (1999a)
	*C. elegans* (Fast killing)	The diversity of toxic molecules produced and released by *P. aeruginosa* facilitates its pathogenicity and contributes to impair lung function in CF.	Cezairliyan et al. (2013)
	Zebrafish embryos	The T3SS, biofilm formation and quorum-sensing systems are involved in virulence, and these systems correlate with increased *P. aeruginosa* virulence in murine models and in humans.	Clatworthy et al. (2009)
	Zebrafish embryos	Helped to support a connection between the cystic fibrosis transmembrane conductance regulator (CFTR) and the innate immune response.	Phennicie et al. (2010)
	*D. melanogaster*	The model allowed *in vivo* study of *P. aeruginosa* biofilm infections.	Mulcahy et al. (2011)
	*G. mellonella* larvae	Identification of mammalian virulence factors of *P. aeruginosa*, particularly biofilm-related genes.	Jander et al. (2000)
*Burkholderia pseudomallei*	*C. elegans*	Disruption of calcium signal transduction, a second messenger in the epithelial response to bacteria, as mechanism for nematode neuromuscular intoxication caused by *Burkholderia spp.*	O’Quinn et al. (2001)
	*C. elegans*	Identification of virulence factors further validated in an intranasal infection model in BALB/c mice.	Gan et al. (2002)
	*D. melanogaster* infected with *Burkholderia thailandensis*	Potential model host to study the role of innate immunity in melioidosis.	Pilátová and Dionne (2012)
*Stenotrophomonas maltophilia*	Zebrafish adults	Abundance of protein Ax21, a quorum-sensing factor, proved correlation to mortality in the zebrafish infection model. This protein triggers innate immunity in both plants and animals.	Ferrer-Navarro et al. (2013)
	*C. elegans* (Slow killing)	Diffusible signal factor (DSF) that controls cell–cell communication, is involved in virulence, biofilm formation, and motilities.	Huedo et al. (2014)
	*G. mellonella* larvae	The model confirms protease StmPr1 as relevant virulence factor of *S. maltophilia.*	Nicoletti et al. (2011)

### ZEBRAFISH (*Danio rerio*) AS AN ALTERNATIVE VERTEBRATE MODEL FOR STUDYING LUNG INFECTION AGENTS

In adult fish, respiration takes place mainly through the gills. In embryo zebrafish gill development begins by 3 days post-fertilization, in the meantime cutaneous respiration accounts for nearly all gas exchange (Rombough, 2007). Naturally, zebrafish get infected by pathogens through the digestive route, the damaged fish surface or through the gills (Cantas et al., 2012). Over the past decade, several bacteria and viruses have been studied in their ability to infect zebrafish (Martin and Renshaw, 2009). A major advantage for its use has been that during the first days after fertilization (≈48 h until hatching) the embryos look transparent and until 3 weeks the larvae are quite translucent (Ali et al., 2011). Therefore, it is possible to follow in real time the progression of infected living embryos, using fluorescent techniques (Redd et al., 2006). However, adult fishes are gaining recognition as a model for bacterial infections because they possess a fully developed adaptive immune system (Meijer and Spaink, 2011).

The second main advantage of this model is the great possibilities that it offers for genomic and large-scale mutant analysis. Zebrafish genome is already available (Ramachandran et al., 2010) and quite well-annotated (ZF version 9; http://www.ensembl.org/Danio_rerio/Info/Index). More than 26,000 genes encoding proteins have been sequenced and annotated, showing high conservation between innate and adaptative related genes with the respective orthologs in humans (Meeker and Trede, 2008; Cui et al., 2011; Rauta et al., 2012).

Most of the mammalian immune system components and molecules have been identified in zebrafish or in other teleost species (Mitra et al., 2010; Alejo and Tafalla, 2011; Xu et al., 2012; Page et al., 2013), including a population of antigen-presenting cells very similar to the mammalian dendritic cells (Lugo-Villarino et al., 2010). Innate immunity is functional, with macrophages and neutrophils that are active at 48 h post-fertilization. These species have an active complement system which can be started /initiated by the same ways presents in mammals (Holland and Lambris, 2004; Sunyer et al., 2005). The adaptive immune system also consists of T cells and B cells although the main site for antigen presentation and T cell maturation is the spleen. Furthermore, multiple waves of hematopoiesis in zebrafish occur at distinct anatomical sites analogous to mammalian hematopoiesis (Kanther and Rawls, 2010).

Renshaw et al. (2007) established a model of inflammation, injuring to the zebrafish tailfin and inducing a characteristic neutrophilic inflammatory response, which resolves with a similar kinetics as in mammals. These authors defined a new model for *in vivo* study of inflammation resolution and their link with apoptosis (Renshaw et al., 2007). By other way, Benyumov et al. (2012) developed an *in vivo* zebrafish model to test phenotypic differences between human fibroblasts that participate in physiological and pathological process.

Additionally, zebrafish has been considered as replacement method for animal experiments because they present some characteristics such as high rate of fecundity, small size, easy maintenance, fast development and less stringent regulatory and ethical considerations since it has been considered that fish embryos in early developmental stages do not experiment pain, suffering, or distress. Although the ethical constraints become apparent, one study suggests that experiments with zebrafish should be subject to regulation from 5 days post-fertilization onward (Strähle et al., 2012) since is between days 5 and 6 when larvae start to feed. Thus, an animal protocol should be required to infect zebrafish older than the time the animals become free feeding. On the other hand, some recent studies say that common anesthetics are not the most “human” or humanize option for zebrafish euthanasia and could cause animal suffering (Cressey, 2014), adding evidences that it is necessary to minimize distress or death.

**FIGURE 1:**
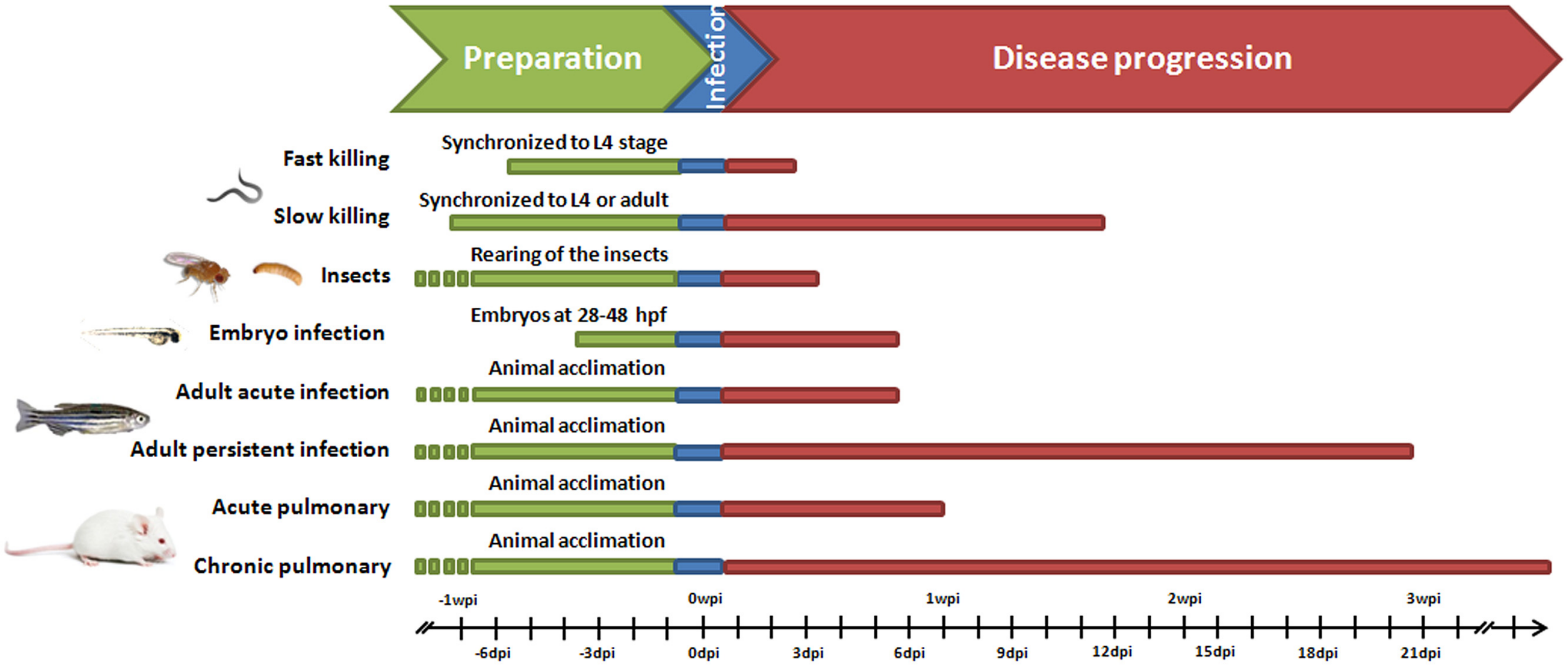
**Time course of implementation of mice and alternative models for bacterial infections**. Persistent infection in adult zebrafish refers to a latent disease Mycobacterium marinum model (Parikka et al., 2012).

As in preclinical researches with mammalian models, adult animals should be allocated for several days under special conditions before beginning the experimental procedures, in order to reach their adaptation to new laboratory conditions and to recover from stress (**Figure 1**). When zebrafish embryos are used, this time is relatively short because they develop very rapidly. To reach a pathogenic dose, as for traditional infection in mice models, zebrafish infection involves a single dose of bacteria requiring an initial population of pathogens able to proliferate avoiding, long enough, the detection by immune cells. However, when adult fish are less susceptible hosts for bacterial infection, higher amount of live bacteria are required when compared to infection in mice. For instance, intraperitoneal infection of zebrafish with the *Pseudomonas aeruginosa* PAO1 strain or with *Stenotrophomonas maltophilia* clinical isolates reported median lethal dose (LD_50_) values of approximately 5 × 10^7^ cfu/dose or 5 × 10^8^ cfu/dose respectively (Ferrer-Navarro et al., 2013; Huedo et al., 2014; Ruyra et al., 2014). Infection models in zebrafish usually are conducted within the time frame of days rather than weeks to months (**Figure 1**), but in the adult persistent mycobacterial infection model a timeline of progression of infection could reach to weeks post-infection (Cronan and Tobin, 2014; Hammarén et al., 2014). Also in zebrafish embryos and larvae, *P. aeruginosa* requires higher dose of pathogens to establish a virulent infection because many of these pathogens are killed by macrophages and neutrophils (Brannon et al., 2009).

### *Caenorhabditis elegans* AS A NON-VERTEBRATE MODEL FOR STUDYING OF LUNG BACTERIAL AGENTS

In 1963 Sydney Brenner proposed the nematode *C. elegans* as an experimental organism for pursuing research in developmental biology and neurology (Ankeny, 2001). *C. elegans* has fewer than 1000 body cells when completely grown. *C. elegans* possesses remarkable advantages that make it an ideal model, for example, low cost, simple growth conditions, and a short generation time with an invariant cell lineage. By other way, at present, there are many molecular and genetic methods for its manipulation (Kurz and Ewbank, 2000). The genome of *C. elegans* was completely sequenced at the end of 1998 (Schulenburg and Ewbank, 2007). In comparison with mice, is obvious that culture and maintenance of *C. elegans* is far simpler and cheaper. Besides, ethical regulations are practically absent for experimentation with worms. Regarding to cell cultures, *C. elegans* contaminated stocks are easily identified and cleaned better than mammalian contaminated cells (Hulme and Whitesides, 2011).

Fascinatingly, this worm shares similarity to mammalian immune system, particularly signaling cascades in innate immunity in response to pathogen invasion (Alper et al., 2008). Because nematodes consume microorganisms as their food source, there is presumably selection pressure to evolve and maintain immune defense mechanisms (Kim et al., 2002; Alper et al., 2010; Shivers et al., 2010). While the human lung and the nematode intestine clearly differ in several anatomical, cellular and biochemical characteristics, in the nematode intestine it is possible to identify pathogen-specific virulence factors that interact with epithelial surfaces. To reinforce the wide field of applications of such non-vertebrate models, the study carried out by Green et al. (2009) describe, despite of the absence of lungs in the nematode, a practical model to evaluate the impact of the cigarette smoke exposure on innate immunity. In this study, *C. elegans* responded to nicotine, which is one of the main components of cigarette smoke, converting nicotine to cotinine, in a similar manner to mammals and opening the path to demonstrate that the animals are absorbing the smoke. In this model, *C. elegans* was subjected to whole cigarette smoke exposure, overcoming several aspects impossible to elucidate using human epithelial cell lines, showing down-regulation in many genes in response to the smoke, mainly several host defense genes (Green et al., 2009).

Additional advantage of using nematodes as animal model of bacterial infection lies in reduced experimental time without the need of animal acclimation before infection, and time-kill curves taking only few hours (fast killing) or days (slow killing) post-infection depending on the killing mechanism (**Figure 1**). However, nematodes need to be synchronized about 1 week prior to infection to reduce the variation in results associated to differences of age. There are two methods to synchronize worms: egg preparation via bleaching and egg lay (Sulston and Hodgkin, 1988). The first method produces more progeny than the latter; however, egg lay can generate a better synchronized population. Scientists take also advantage of temperature-sensitive sterile *C. elegans* mutants (e.g., the CF512 strain) to avoid production of a brood that would complicate the scoring of death of the infected worms. These animals do not produce progeny at working temperature (25°C), thus simplifying the procedure. However, these strains appear to be slightly more resistant to bacterial infections (Feinbaum et al., 2012).

More than 40 human pathogens, or their close relatives, are known to cause disease in *C. elegans* (Sifri et al., 2005). *P. aeruginosa* was the first broad host-range pathogen, able to kill *C. elegans* (Tan et al., 1999a,b). *C. elegans* infection process has the advantage to closely resemble chronic infection, because the host is usually exposed to maximal dose of pathogen. On the other hand, among killing mechanisms, slow killing involves a process similar to those related to an infection process. At present, there are five distinctive mechanisms of worm killing identified: infection with intestinal colonization, persistent infection, invasion, biofilm formation, and toxin-mediated killing (Smith et al., 2002; Beale et al., 2006; Dunbar et al., 2012). Obviously, in the *C. elegans* model will be possible to study only those human diseases caused by pathogens able to infect the nematodes. In addition to the bacterial species, the killing mechanism is in most cases dependant to the way the bacterium is prepared prior to infection. For instance, depending on the composition of the agar medium where the bacterium is grown, the rate of *P. aeruginosa*-mediated killing of *C. elegans* will be different (Tan et al., 1999a). If the *P. aeruginosa* tested strain is grown on minimal medium, the killing will occur in the course of several days; by the contrary, if a high-osmolarity medium is used, the killing will occur in the course of several hours.

### INSECT AS MODELS OF INFECTION

In opposite to mammals, the respiration process in insects occurs by a network of tubes called tracheae and tracheoles. Despite this, insects have recently been shown to be a valuable alternative to animal models for bacterial pathogenesis studies. This is mainly due to the fact that insects have a relatively advanced system of antimicrobial defenses. Like mammals, insects possess a complex innate immune system and display evolutionary conservation of signaling cascades (Lemaitre and Hoffmann, 2007). In addition, as with other non-vertebrate models, the advantages are the low cost of maintenance and no ethical concerns. There are multiple genetic and molecular tools available. By other way, these models have a precise endpoint. The low cost of maintenance and the rapid development, allow their use in high animal number for proper statistical analysis of results. Among these models, the common fruit fly *D. melanogaster* and the larvae of *G. mellonella*, have been shown to be relevant for several fungal and bacterial mammalian pathogens (Limmer et al., 2011; Sprynski et al., 2014).

Most insects have a very rapid life cycle, which consists of four clearly defined stages: the embryo, the larva, the pupa, and the adult. In addition, insect rearing is easy and relatively cheap (Ramarao et al., 2012). For larva, drugs can be administered directly injecting the organism or mixing with media (solid or liquid with 2% yeast paste). For adults, drugs may be delivered as aerosol, mixed with food, injected or applied directly to the nerve cord. In the injection method, a needle or a nanoinjector preloaded with pathogen culture is used to prick the body cavity (insect hemocoel). Injection requires anesthetization, which is usually done with carbon dioxide, and requires the transfer of insects into vials containing food, where the worms incubated at 25–30°C and their survival is evaluated (Igboin et al., 2012). For ingestion, it is common to introduce the insects into small laboratory tubes containing filter disks embedded with media containing pathogens of interest.

*Galleria mellonella* larvae are cost effective, widely available and the results can be obtained within 2 or 3 days (**Figure 1**). There are three main ways in which *Galleria* fight bacterial infections: circulating phagocytic hemocytes that patrol the hemolymph; proteolytic cascades that can be quickly triggered, activating the melanization response and inducing antimicrobial immune effectors such as lysozymes, as well as antimicrobial peptides which can be rapidly synthesized by the fat body (Yuen and Ausubel, 2014). An added benefit of using *Galleria* for pathogenesis studies is that infections can be carried out at 37°C or higher, as *Galleria* tolerates relatively high temperatures, unlike the Zebrafish, *D. melanogaster* and *C. elegans* (maximum 25–28°C; Glavis-Bloom et al., 2012). The larger size of the *Galleria* larva, compared to other invertebrate models, also allows it to be infected with larger and more controlled doses of the pathogen without significantly traumatizing the insect. Some disadvantages of the *G. mellonella* model rely in the fact that genetic methods for generating recombinant organisms and to sequence them are not completely available. However, this model could be improved in the next years and hopefully, could be used for more pathogens, for which no alternative models of infection exist.

## FROM CLASSIC TO ALTERNATIVE MODELS IN STUDYING RELEVANT BACTERIAL LUNG PATHOGENS

The most common causes of bacterial lung infections in normal human hosts include *Streptococcus pneumoniae*, *Haemophilus influenzae* and *Staphylococcus aureus*, and the recent increase of *M. tuberculosis*. Pneumonia is classified according the source of infection into community-acquired pneumonia (CAP), hospital-acquired or nosocomial, aspiration of foreign material and immunocompromised host (Woodhead, 2013). In basis of their presentation, pathogens have been classified into “typical” and “atypical.” Typical organisms in CAP include *S. pneumoniae*, *H. influenzae*, *S. aureus*, *Moraxella catarrhalis,* and *P. aeruginosa* (Musher and Thorner, 2014). Atypical organisms include *Legionella* species, *Mycoplasma pneumoniae, Burkholderia spp.*, *Chlamydia spp*., *Chlamydophila spp.*, *Coxiella burnetii* and viruses (Jones, 2010). In almost all epidemiological studies of hospital-acquired pneumonia (Jones, 2010; Barbier et al., 2013; Polverino et al., 2013; Erdem et al., 2014), a consistent six organism groups (*S. aureus*, *P. aeruginosa*, *Klebsiella* species, *Escherichia coli*, *Acinetobacter* species, and *Enterobacter* species) caused ∼80% of episodes, with lower prevalences of *Serratia* species, *S. maltophilia*, and community-acquired pathogens, such as pneumococci and *H. influenzae*. In compromised hosts, the bacterial causes of pneumonia are much broader, including species not usually considered of high virulence in humans. For instance, *Mycobacterium sp.*, *Burkholderia spp.*, *P. aeruginosa,* and *S. aureus* are the most important infectious agents in cystic fibrosis (CF) patients (Coutinho et al., 2008).

**FIGURE 2:**
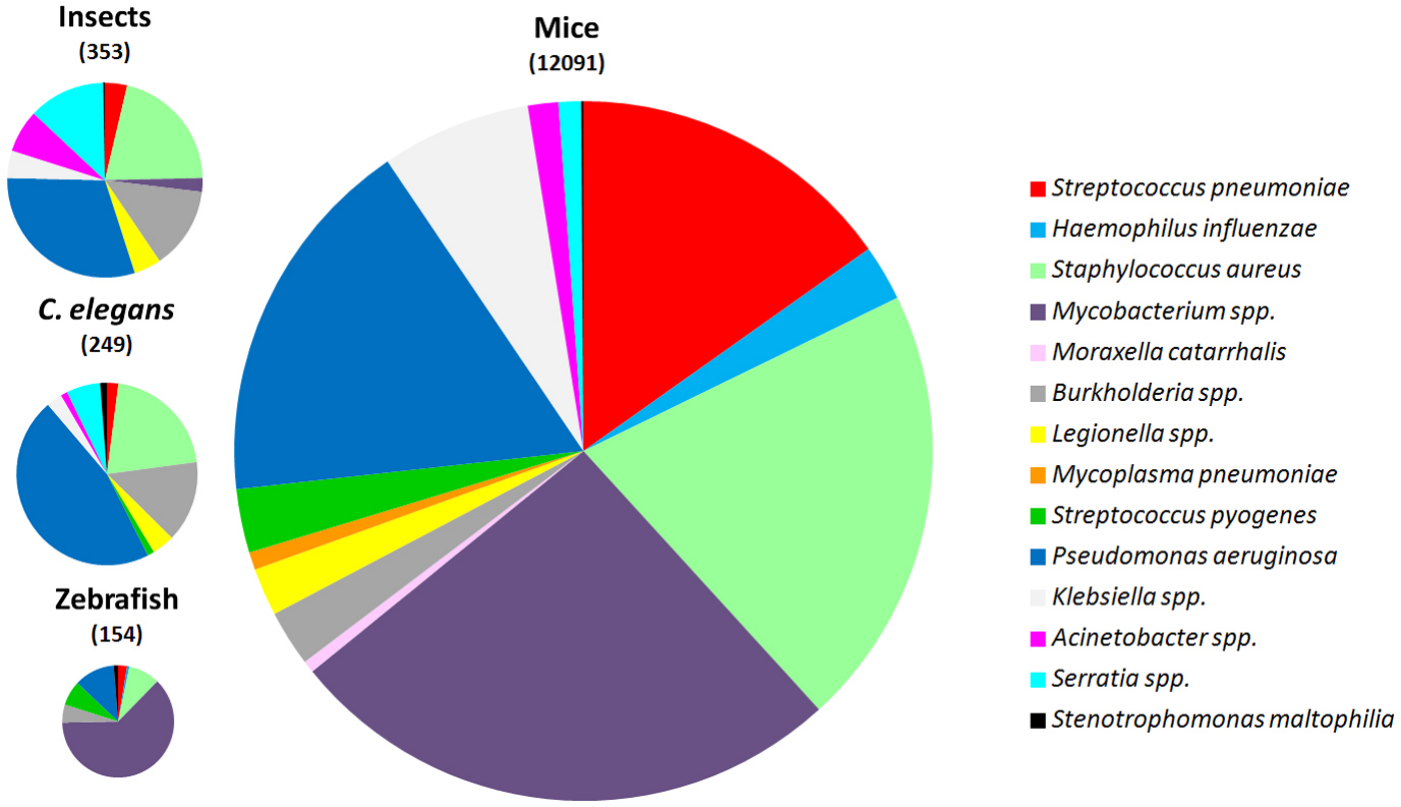
**Comparative number of publications for different lung pathogens employing mice, *Caenorhabditis elegans*, zebrafish, and insects as animal models of infection.** The number of publications is shown between parentheses. A systematic search was conducted using PubMed (http://www.ncbi.nlm.nih.gov/pubmed). Literature searches were conducted to identify studies published until 1 November, 2014.

For the most frequent bacteria causing pneumonia, scientists have developed animal models of infection, mainly using mice (**Figure 2** and **Table 1**). However, the introduction of alternative non-mammalian models is still at its beginning and obviously for host-permissive pathogens the contribution would be higher. For instance, despite several mouse *M. tuberculosis* lung infection models are utilized, and *Mycobacterium marinum* infection of fishes results in chronic granulomatous diseases similar to mycobacterioses in mammals (Cosma et al., 2006b), *C. elegans*, a well-established model host, is resistant to mycobacterial infection (Couillault and Ewbank, 2002). The most extreme example is that of pathogens for which there are no or very few alternative models of infection, such as *H. influenzae*, *M. catarrhalis,* and *M. pneumoniae* (**Figure 2**). On the other side as will be discussed later, *P. aeruginosa*, a versatile and ubiquitous bacterium, is capable to survival and colonize various living host organisms facilitating the development of infection models spanning from nematodes to small vertebrates (**Figure 2**). Here, we discuss the models that have been developed for studying most common human lung pathogens by comparing the mouse model with alternative ones in zebrafish, nematodes, and insects.

### ANIMAL MODELS OF PNEUMOCOCCAL INFECTION

*Streptococcus pneumoniae* is considered the most common bacterial agent in CAP and a great number of animal models of pneumococcal diseases are available. Some review articles from the past decade have specifically mentioned the value of animal models to test pneumococcal vaccines (Briles et al., 2000; Adamou et al., 2001; Bogaert et al., 2004; Kadioglu and Andrew, 2004; Morsczeck et al., 2008) and the use of murine models of pneumonia to evaluate protein-mediated antimicrobial responses (Srivastava et al., 2007), the mouse genetic susceptibility to pneumococcal disease (Proft and Fraser, 2003) and for investigating the pathophysiology of bacterial meningitis (Teles et al., 1997; Gerber et al., 2001; Blair et al., 2005; Endo et al., 2012).

On the other hand, as there are several evidences about the positive correlation between infection with *Streptococcus iniae* and *Streptococcus pyogenes* in zebrafish and mammalian models, there is simple to assume the use of zebrafish to evaluate host–pathogen interactions during pneumococcal infection (Borst et al., 2013). In a recent work, Rounioja et al. (2012) reported the use of zebrafish embryo to evaluate the response of the host immune system against challenge with pneumococci. They also reported that this response is dependent on whether the pneumococci could evade clearance by interfering with host phagocytic function. Moreover, pneumococcal mutants defective in important virulence factors were attenuated in this *in vivo* model system (Rounioja et al., 2012). The adult zebrafish model can be also used to investigate pneumococcal diseases (Saralahti et al., 2014). The authors showed that *S. pneumoniae* mutants defective in polysaccharide capsule are also attenuated and that elimination of pneumococci depends mainly on host innate immune responses.

To our knowledge, little is the information available up to day related to the use of *C. elegans* to study pathogenesis of *Streptococcus*. Garsin et al. (2001, 2003), in two separate works, demonstrated the suitability of using *C. elegans* as a model host for Gram-positive infection, including *Enterococcus faecalis*, *S. aureus*, and *S. pneumoniae*. Jansen et al. (2002) demonstrated that *S. pyogenes* kills *C. elegans*, both on solid and in liquid medium, mediated by the hydrogen peroxide production (Jansen et al., 2002; Bolm et al., 2004).

The potential application of the larvae of *G. mellonella* as an informative infection model for *S. pneumoniae* has been also studied (Evans and Rozen, 2012) since strains differing in known virulence factors could be distinguished in this host. Strains lacking capsule or pneumolysin showed less virulence than their respective wild types. Particularly, pneumolysin plays a role in damaging human lung epithelium, allowing the establishment of infection (Rayner et al., 1995).

### ANIMAL MODELS OF STAPHYLOCOCCAL INFECTION

As nasal colonization is a main requisite for the establishment of *S. aureus* infection in humans (Baur et al., 2014), mice infected by using this route is a useful model for characterizing early host responses. However, this model has failed in mimic the whole natural route of infection, resulting in self-limited disease (Alami et al., 1968; Bartell et al., 1968; Anatoliĭ et al., 1971; Ansfield et al., 1977; Bragonzi et al., 2004; Bloemendaal et al., 2011). The Bolus infection models, where mice are challenged by i.t. and i.n. inoculation have been more successful in producing intrapulmonary infection and host mortality (Jakab, 1976; Hu et al., 2006; Rajagopalan et al., 2006; Huzella et al., 2009; Fernandez et al., 2011; Park et al., 2011). Sawai et al. (1997) described a murine model of acute staphylococcal pneumonia inoculating mice by intravenous (i.v.) injection of a suspension containing bacteria enmeshed in agar-beads. This model allows the bacteria to remain in the lung for several weeks, and it is reproducible and simple (Sawai et al., 1997).

Li and Hu (2012) developed a zebrafish embryo infection model with *S. aureus* at 36 h post-fertilization. These researches inoculated the bacteria directly into the pericardial cavity, eye, and yolk body. By using GFP-expressing *S. aureus* and transgenic zebrafish lines along with multicolored confocal fluorescence methods, they could analyze different phases of bacterial infection. As important conclusion from this work, the dynamic of infection clearly depends on the bacterial entry routes (Li and Hu, 2012). Prajsnar et al. (2008), using a similar model, identified staphylococcal virulence genes, whose respective mutants are attenuated in zebrafish. The virulence factors include a peroxide regulon repressor, a protein involved in starvation survival and a response regulator involved in controlling exoproteins production. They also demonstrated that in zebrafish embryos, macrophages phagocytize *S. aureus* during *in vivo* infections. Accordingly to Kubica et al. (2008), these immune cells act as a reservoir during infection. This model in both zebrafish embryos and adults also allowed rapid screening of mutants for those strains with attenuated pathogenicity, identifying relevant factors of pathogen virulence and host immunity (Lin et al., 2007; Li and Hu, 2012; Lü et al., 2012).

On the other hand, several studies used the *C. elegans* model to assess virulence levels between some different methicillin-resistant *S. aureus* strains, demonstrating the suitability of this model for studying the virulence and pathogenicity of *S. aureus* strains (Wu et al., 2010, 2012, 2013). In addition, several *S. aureus* virulence determinants recognized as important in mammalian pathogenesis are also identified as relevant for full pathogenicity in nematodes, including *agr* (a quorum-sensing global virulence regulatory system), *sarA* (global virulence regulator), the alternative sigma factor B, alpha-hemolysin, and V8 serine protease (Sifri et al., 2003, 2006). However, Polakowska et al. (2012) showed that there is no substantial variation in virulence among different staphylococcal strains using this experimental model, questioning the usefulness of it.

In another work, JebaMercy et al. (2011) performed solid and liquid assays for the infection of *C. elegans* with *S. aureus*, demonstrating that *S. aureus* took ∼90 h for the complete killing of *C. elegans* and thereby postulating that colonization with live bacteria was necessary for worm killing. Using an interactive genetic approach, Begun et al. (2007) established a novel *in vivo* experimental model to explain the interaction between the bacteria biofilm matrix and components of the innate immune system. This study demonstrates the ancient conserved function against predation linked to the protective activity of biofilms. In another work based on *C. elegans* infections, the same authors identified staphylococcal genes relevant for mammalian pathogenesis, including the product of the *nag*D gene, which was not previously described as a virulence factor (Begun et al., 2005).

Larvae of the greater wax moth also have provide insights into the pathogenesis of *S. aureus*, principally as a suitable host for testing the *in vivo* efficacy of antimicrobial agents (Gao et al., 2010; Desbois and Coote, 2011; Gibreel and Upton, 2013; Apolónio et al., 2014). In addition, using this models authors have demonstrated for the first time that both glycolysis and gluconeogenesis have important roles in virulence (Purves et al., 2010). Their results showed that two glyceraldehyde-3-phosphate dehydrogenase (GAPDH) homologs (GapA and GapB) are required for the virulent phenotype of *S. aureus* in this model. In *S. pyogenes* surface-associated GAPDH was associated with antiphagocytic properties and host cell adherence (Boël et al., 2005).

### ANIMAL MODELS FOR MYCOBACTERIAL PATHOGENESIS

Due to the complex interaction between the pathogen and the host, it has been very difficult to find out an ideal model to study mycobacterial pathogenesis. Some mice strains can be easily infected via aerosol with a low dose of *M. tuberculosis*, multiplying in the lungs and subsequently spreading to liver and spleen. The infection is controlled but not eliminated, by cell-mediated immunity, mainly T cell responses, and the infection is well-tolerated for more than 1 year (Beamer and Turner, 2005; Aguilar et al., 2007, 2010; Andreevskaia et al., 2007). Hence, the mouse model has been largely a suitable infection model (Orme, 2005b; Cooper, 2014). Surprisingly, there is still no ideal and validated model of experimental tuberculosis disease (Vilaplana and Cardona, 2014), and the mechanisms leading to latency and reactivation of are still unclear (Parikka et al., 2012).

BALB/C mice infection models by i.t. injection using a high dose of bacilli (Hernández-Pando et al., 1996) is one of the most employed models. This model has greatly contributed to elucidate the role of antibodies in the protection against mycobacterial infections (López et al., 2009, 2010; Alvarez et al., 2013), and to the screening and validation of new vaccine candidates (Castillo-Rodal et al., 2006; López et al., 2006). Alternatively, C57BL mice have been infected i.n. via aerosol with a low dose of *M. tuberculosis*, which produces a well-tolerated infection dominated by Th1 response (Kelly et al., 1996; Cardona et al., 2003; Aldwell et al., 2005; Saini et al., 2012). For this reason, this is actually a model of slow progressive disease, and the animal death is produced by excessive inflammation or immunopathology (Mustafa et al., 1999a,b, 2000). The model of latent tuberculosis has been also partially reproduced experimentally in mice (Phyu et al., 1998; Shi et al., 2011; Zhang et al., 2011) which are injected intratracheally with relatively low numbers of the virulent strain H37Rv (Hernandez-Pando et al., 1998). After that, low and stable bacillary counts with few granulomas appear and the mice continue gaining weight and appear healthy for more than 2 years.

Although mice are the most employed animal model for studying human TB, it has important drawbacks. Due to the fact that *M. tuberculosis* is not a natural pathogen of mice, the pathological development of TB will be clearly different from that in human. As relevant characteristic, we can mention the absence of granulomas formation in lungs from mice (Grumbach et al., 1967; Karakousis et al., 2004; Mollenkopf et al., 2004; Orme, 2005a,b; Hunter et al., 2007; Dharmadhikari and Nardell, 2008; Young, 2009; Apt, 2011). The immune response elicited in mice after mycobacterial infection is able to control bacillary load even without causing marked lesions (Vilaplana and Cardona, 2014). Therefore, mice are generally resistant to TB infection when compared with other rodents, and even humans, as evidenced by their ability to tolerate relatively large bacterial numbers within their lungs without signs of disease (Be et al., 2008; Dharmadhikari and Nardell, 2008; De Steenwinkel et al., 2009). Also, ethically, these models in conjunct appear to be more aggressive to the mice.

Zebrafish and fruit fly are emerging as alternative models and have provided new insights into the pathogenesis of the tuberculosis disease. By the contrary, *C. elegans*, to our knowledge, seems to be not a feasible model for infection with *M. tuberculosis*. The zebrafish has been a key model in our understanding of mycobacterial infection. Studies on this model employ a fish pathogen, *M. marinum*, a close relative to *M. tuberculosis* (Tobin and Ramakrishnan, 2008). This bacterium is a natural pathogen of fish and amphibian (van der Sar et al., 2004a,b). The *M. marinum* infection produced in these hosts is quite similar to those produced in humans, mainly in the granuloma formation (Prouty et al., 2003; Berg and Ramakrishnan, 2012). Low doses (<100 bacteria) of *M. marinum* lead to a chronic infection in adult zebrafish (Parikka et al., 2012), while higher doses cause a fatal acute infection (Cosma et al., 2006b). Besides, *M. marinum* grows faster than *M. tuberculosis*, it can be easily manipulated and requires only common laboratory precautions (biosafety level 2). The zebrafish/*M. marinum* infection model changed the old conception that granuloma formation requires lymphocytes and by the contrary, postulated that the granuloma actually functions as a bacterial tool for disseminating the disease.

Experiments conducted by Ramakrishnan and colleagues using the zebrafish/*M. marinum* infection model (Davis et al., 2002; Davis and Ramakrishnan, 2009; Ramakrishnan, 2013), demonstrated for the first time new mechanisms of bacterial dissemination: the bacterial transfer between two macrophages through membrane tethers and re-phagocytosis of bacteria associated with dead macrophages in tissues. They also observed that, as early as 72 h post-injection, the extravasated infected macrophages began to form granuloma-like aggregates in the tissues, establishing that there is not needed the participation of adaptive immunity to initiate granuloma formation (Davis et al., 2002). Using this model, and taking advantages of the powerful live imaging of the zebrafish model, it was determined that an efficient bacterial expansion depends on the mycobacterial region of deletion 1 (RD1) locus. The researchers also demonstrated that the bacterial protein ESAT6 elicits the expression of metalloproteinase 9 (MMP9) in the host, and both proteins act together for granuloma formation (Pozos et al., 2004; Volkman et al., 2004; Cosma et al., 2006a; Berg and Ramakrishnan, 2012; Takaki et al., 2013; Cambier et al., 2014). These findings showed that one protein from the host and one from the bacteria, constitute a virulence axis which evade the host’s early immune responses and lead to mycobacterial dissemination. This study is, from our point of view, the most important evidence of how the zebrafish model can be used to validate and to re-postulate host–pathogen interactions during mycobacterial infection.

On the other hand, Swaim et al. (2006) reported that lymphocytes play the same critical role in controlling mycobacterial infection in fishes and mammals by the use of a defective zebrafish mutant in the *rag1* gene. They also demonstrated that bacteria defective in RD1 region are also attenuated in zebrafish. In addition, the zebrafish/*M. marinum* model has proven to be useful for studying the latency, dormancy, and reactivation of latent or subclinical tuberculosis (Parikka et al., 2012). This group has recently studied using this model the T cell responses in mycobacterial infection and they have found associations between the disease severity (bacterial load) and the type and magnitude of T cell responses, particularly an adequate Th2-type response (Hammarén et al., 2014). This infection model also helped to demonstrate that mycobacterial antigens Ag85B, CFP-10, and ESAT-6 protect adult zebrafish from mycobacterial infection (Oksanen et al., 2013), paving a new way in tuberculosis vaccine research.

*Mycobacterium marinum* also causes a lethal infection in the fly *D. melanogaster* characterized by a widespread tissue damage, even at significant low bacterial doses (Dionne et al., 2003). These initial stages of the infection were very similar to the early stages in frogs and fishes infected with *M. marinum* (Pozos et al., 2004). This model may be valuable in testing the activity of new antimycobacterial agents (Oh et al., 2013).

### ANIMAL MODELS TO STUDY THE VIRULENCE FACTORS OF *Pseudomonas aeruginosa*

*Pseudomonas aeruginosa* is an opportunistic human pathogen that can also infect several diverse organisms, such as plants, nematodes, insects, and mammals. Thus, we counted on good mammalian and non-mammalian models for studying virulence factors of *P. aeruginosa*. In humans, *P. aeruginosa* is widely associated with nosocomial infections in CF patients (Moreau-Marquis et al., 2008) and other immunocompromised individuals, and the resolution of the infections is hampered due to the formation of drug resistance biofilms. Until the moment, a difficult exists on studying biofilms formation in the context of animal and human lungs. However, several non-mammalian models have provided compelling data regarding *P. aeruginosa* biofilm formation (reviewed in Lebeaux et al., 2013).

Acute and chronic models of lung *P. aeruginosa* infection have been developed using several mammalian species (rats, guinea pigs, hamsters, mice, sheep, rabbits, and baboons; Seidenfeld et al., 1986; Starke et al., 1987; Pier et al., 1990; Collins et al., 1991; Iwata and Sato, 1991; Hart et al., 1993; Terashima et al., 1995; Bakker-Woudenberg et al., 2002; Luna et al., 2007, 2009; Kurahashi et al., 2009; Rodríguez-Rojas et al., 2009; Collie et al., 2013). The chronic infection model has been extensively used and characterized, showing certain similarities with human pathology due to the persistence of the inoculum and the resultant lung pathology (van Heeckeren and Schluchter, 2002). Depending on the route, dose administered, and the frequency of dosing, acute lung infection with either rapid clearance of the bacteria or acute sepsis and death could take place (George et al., 1991). Using this model, it has been shown that *P. aeruginosa* must express several key virulence factors (Balloy et al., 2007).

A literature survey about acute vs. chronic *P. aeruginosa* lung infections clearly shows that to induce an infection for more than 1 month, it is necessary to use an immobilizing agent such as agar, agarose, or seaweed alginate together with the bacterial suspension (Iwata and Sato, 1991; Hart et al., 1993; McMorran et al., 2001; Moser et al., 2002). The initial agar-beads model of chronic pulmonary infection with *P. aeruginosa* was modified for its use in mice by Starke et al. (1987) and has been widely used to study CF lung disease, bacterial pathogenesis (Gosselin et al., 1995, 1998; Morissette et al., 1995; Stevenson et al., 1995; Moser et al., 1997; Sapru et al., 1999; Tam et al., 1999; McMorran et al., 2001), and for the evaluation of new therapies (Pier et al., 1990; Staczek et al., 1998; Chmiel et al., 1999; Wilmott et al., 2000) and virulence factors (Rodríguez-Rojas et al., 2009). Researchers have also developed a mouse model with a lung pathology similar to human CF. One group generated mice that absorb excess sodium in the airways (Mall et al., 2004; Mall, 2008) and these animals developed airway obstruction with dehydrated mucus. This CF model promises to answer important questions about the cause of the inflammation that leads to lung damage and failure in CF (Mall et al., 2004; Mall, 2008). Both acute and chronic models require extensive use of animals and labor-exhausting techniques to prepare and immobilizing bacteria in agar, as well as good surgery skills.

Brannon et al. (2009) developed a zebrafish embryo infection model for the study of systemic *P. aeruginosa* infection, and for evaluating the virulence of a type 3 secretion system (T3SS) mutant. By fluorescence microscopy it was possible to follow in real time *P. aeruginosa* infection in transgenic zebrafish with fluorescently labeled neutrophils and macrophages (Hall et al., 2007). Clatworthy et al. (2009) demonstrated that lethal infection requires quorum-sensing and the T3SS for full virulence in late-stage zebrafish embryos infected with *P. aeruginosa*. Curiously, T3SS expression has been associated also to increased risk of *P. aeruginosa* infection in hospitalized patients (Ledizet et al., 2012) and it is also associated with initial infections in patients with CF (Jain et al., 2008). They demonstrated that the infection outcome could be influenced on the pathogen side, by both the inoculum size and the presence of known virulence determinants (*las*R, *mvf*R, and *psc*D) and on the host side by developmental stage and the modulation of the immune system (Clatworthy et al., 2009). They also showed that the infection process can be modified through the use of morpholinos or antibiotics, which were used to shift immune cell numbers or rescue embryos from lethal challenge respectively.

In one study performed by Phennicie et al. (2010) in zebrafish, the role of the cystic fibrosis transmembrane conductance regulator (CFTR) in the innate immune response to acute infection with *P. aeruginosa* was evaluated. The authors found that the *P. aeruginosa* bacterial load was significantly higher in *cftr* morphants (knockdown of the zebrafish ortholog to human *cftr*) than in control embryos, according with similar studies performed with mice and human bronchial epithelial cells.

The *P. aeruginosa*-zebrafish infection model has allowed conducting chemical screens for small molecules or antimicrobial compounds. For instance, the treatment of infected embryos with front line antipseudomonal agents could save zebrafish embryos from a lethal *P. aeruginosa* challenge (Clatworthy et al., 2009). More recently, Ruyra et al. (2014) reported the use of immunostimulant-loaded nanoliposomes to protect adult fishes against bacterial or viral infections. In a model of adult zebrafish infection developed by these researchers, nanoliposomes protected zebrafish against otherwise lethal bacterial (*P. aeruginosa* PAO1) and viral (SprinViraemia of Carp Virus) infections.

Tan et al. (1999a; 1999b) conducted several studies (Tan and Ausubel, 2000; Tan, 2002) showing for the first time the use of *C. elegans* in the study of *P. aeruginosa* pathogenesis. They demonstrated that accumulation of *P. aeruginosa* cells in intestines is crucial to explain the killing mechanism. In addition, as other authors have demonstrated, they showed that bacterial genes required for this killing were also described in mammalian or plant hosts pathogenesis. Feinbaum et al. (2012) screened mutants with reduced ability to kill *C. elegans* using a mutant library representing ∼80% of the non-essential *P. aeruginosa* PA14 genes. They described a set of 180 *P. aeruginosa* genes necessary for normal levels of virulence. The principal contributors to *P. aeruginosa* virulence in the *C. elegans* infection model were genes that play key roles in survival of *P. aeruginosa* within the host intestine, particularly regulatory genes that are involved in quorum-sensing (Feinbaum et al., 2012).

Insects have been also surrogate model systems for identifying mammalian virulence factors of *P. aeruginosa*. Previous studies showed that this bacterium is a virulent pathogen of fruit flies (Boman et al., 1972). Using the *D. melanogaster* as model host, D’Argenio et al. (2001) have identified mutants of *P. aeruginosa* with reduced virulence. Among these mutants, the *pil*-*chp* signal transduction system is particularly relevant also in mammals and is involved in type IV pilus synthesis and biofilm formation (Kato et al., 2008). The *D. melanogaster* model allowed *in vivo* study of *P. aeruginosa* biofilm infections by oral administration (Mulcahy et al., 2011). By the other hand, several studies summarized by Jander et al. (2000) point out similarities between virulence of *P. aeruginosa* mutants in mice and *G. mellonella*. This infection model helped to demonstrate that human anti-microbial peptides that inhibited the initial steps in biofilm formation could be used in the development of new therapies for *P. aeruginosa* infection (Dean et al., 2011).

### ALTERNATIVE MODELS FOR TWO LESS COMMON BACTERIAL LUNG PATHOGENS

Previous works suggest a limited invasiveness of *Stenotrophomonas maltophilia* in mice, as indicated by a transient and minimal presence of the bacteria in animal organs after infection. *S. maltophilia* CF strains were shown to cause no mortality in a neonatal mouse model of respiratory tract infection (Waters et al., 2007). Despite this lack of strong invasiveness, mouse models of *S. maltophilia* infection have been useful answering questions about immune response against this pathogen (Brooke, 2012). Additionally, a model of acute respiratory infection in DBA/2 mice inoculated with aerosolized *S. maltophilia* has allowed the study of lung pathology and the mechanisms of infection resolution (Di Bonaventura et al., 2010). However, in this model, most of the animals were able to control the infection in a short time period, even at high doses of virulent inoculums, being the animal weight the best criterion to evaluate the virulence of tested strains (Di Bonaventura et al., 2010; Pompilio et al., 2011). One study also showed bacterial colonization in rat lungs after 7 days post-infection (McKay et al., 2003).

*S. maltophilia* has also been isolated from channel catfish (*Ictalurus punctatus*) with infectious intussusception syndrome (Geng et al., 2010), suggesting that the use of fish as a model to evaluate the pathogenicity and susceptibility of *S. maltophilia* to available antimicrobial agents is adequate. Recently, a model of intraperitoneal infection in zebrafish confirms the attenuation of a *S. maltophilia* collection strains when compared with recent clinical isolates (Ferrer-Navarro et al., 2013), paving the way for new approaches to gain relevant information on pathogenesis of this bacterium. An infection model using *C. elegans* has been proposed for routine screening of *S. maltophila* isolates for pathogenesis (Thomas et al., 2013). In this work the *in vivo* killing efficiency was evaluated by four different methods: classical fast killing assay, filter-based fast killing assay, slow killing assay and virulence assay using heat inactivated bacteria. Moreover, virulence regulation in *S. maltophilia* mediated by a quorum-sensing system has been recently studied in the *C. elegans* and zebrafish infection models (Huedo et al., 2014). In that work, it has been demonstrated that *S. maltophilia* inoculated by intraperitoneal route in zebrafish is characterized by rapid body dissemination. By the other hand, one study using the insect *G. mellonella* suggests the proteolysis as a possible pathogenic mechanism in *S. maltophilia* isolates from CF infections (Nicoletti et al., 2011).

On the other hand, respiratory pathogens like *Burkholderia pseudomallei* has the same type of tropism in mice than that observed in humans, regardless of its acute or chronic output (Stundick et al., 2013). Modeling of experimental melioidosis has been conducted in numerous biologically relevant models including mammalian and invertebrate hosts (reviewed in Warawa, 2010). Non-mammalian models have been explored since the mechanisms of *Burkholderia* virulence may be conserved during evolution from worms to mammals. *Drosophila* and *G. mellonella* have also shown to be useful alternative infection models for *Burkholderia spp.* (Castonguay-Vanier et al., 2010; Wand et al., 2011; Pilátová and Dionne, 2012). Particularly, *Burkholderia thailandensis* is highly virulent in the fruit fly (Pilátová and Dionne, 2012), a closely related organism to *B. pseudomallei* known to be avirulent in humans, thus being a useful model for mammalian melioidosis.

Data shown by O’Quinn et al. (2001) suggest that the disease phenotype observed in nematode after exposure to *B. pseudomallei* may be also valuable for investigating the pathogenesis of these bacteria. *Burkholderia* species are able to cause ‘disease-like’ symptoms and kill the nematode *C. elegans* either by infection or intoxication (O’Quinn et al., 2001; Darby, 2005) or suppressing worm immunity by specific degradation of a GATA transcription factor (Lee et al., 2013). The study of O’Quinn et al. (2001) suggests that the neuromuscular intoxication caused by *B. pseudomallei* is related to a signal transduction mechanism involving calcium. It is well-known that bacterial toxins can increase the content of free calcium (Ca^2+^) in the cytosol of the host (TranVan Nhieu et al., 2004). In this sense, calcium acts as a second messenger in several physiological processes and immune mechanisms. The common respiratory bacterial pathogens, *P. aeruginosa* and *S. aureus*, activate Ca^2+^ fluxes after contact with y epithelial cells from the respiratory tract, activating proinflammatory signaling events (Ratner et al., 2001). The Ca^2+^ fluxes mediate the expression of proinflammatory cytokines and chemokines necessary to recruit leukocytes to the lung and also to initiate modifications in the epithelial junctions to facilitate leukocyte transmigration into the airway lumen (Chun and Prince, 2009). The *C. elegans* system has been used to screen for new virulence factors in *B. pseudomallei*, and selected attenuated bacterial mutants were further evaluated in an intranasal infection model in BALB/c mice (Gan et al., 2002). The results in mice validate positively the use and clinical relevance of *C. elegans* as an alternative model in the screening of virulence factors in *B. pseudomallei*.

## CONCLUDING REMARKS

The nematode was the first invertebrate alternative model described, followed by the larvae and adult fruit fly models (*Drosophila sp.*) and more recently, the wax moth larvae (*G. mellonella*) model. Non-mammalian vertebrates, as fish and amphibians, which are able to mount an adaptive immune response, are now available as excellent tools. These kinds of models are usually criticized as being too distant from human. Several limitations such as their reduced complexity and the simplicity of their immune system, differences in temperature, target organs, or particular receptors have impaired the use of these models. However, these have been mentioned also for the murine models of other diseases. No model is perfect, each one has its specific strengths and weaknesses, but the most important thing is to combine the information gained from one to other, taking advantages of the incredible genomics and bioinformatics tools on-hand, before to extrapolate to humans beings. The belief that vertebrates are necessary the best available models in biomedical research was called the high-fidelity fallacy (Stevens, 1992; Russell, 1995). To avoid some of the limitations of these models, researchers begin to study infection and immunity in non-mammalian models. Since our knowledge of the immune system and their evolutionary conservation has increased, the usefulness of alternative models different for mammals has been accepted more. Although could seems irrational to study lung diseases in animals who do not have lungs, several evidences support the benefits that these studies, if carried properly, may to bring in the elucidation of human lung pathology diseases (**Table 1**). Thus, these non-mammalian organisms have been successfully employed to elucidate conserved and universal immune mechanisms. In addition, the small size of the most used non-mammalian organisms enables to perform high throughput and automated studies (Letamendia et al., 2012). Most of these model organisms have their genome completely sequenced, offering the possibility to do genetic studies both on the bacteria and the host. And what we consider one of the most practical advantages, the alternative models described here provide a way to easily bypass the ethical limitations of some types of studies in higher animal models.

What we do recommend to the scientific community facing the design of experimental infection with bacterial pathogens? Firstly, we have to consider several aspects related to the immunopathology of the diseases that we want to reproduce in an animal model. The relationship between the host and the guest in terms of molecular interactions is crucial to determine which type of response we will observe and consequently, to plan strategies for measuring it. But, a question arises. Which, among the methods that we will employ, are better in terms of cause less injury or damage to the animal? Is this response only measurable in vertebrate animal models? There are now sufficient evidences about the similarities of non-vertebrates immune systems and mammals. By other way, it has been demonstrated that the handling and maintenance of non-mammals organisms is easier and shaper. It is common to think in mice immediately when we are planning an *in vivo* experimental infection. Certainly, it is the most employed animal model in biomedical research. However, we could be aware about ethical restrictions that the use of large amount of animals and certain experimental procedures could implicate for the investigation. So, we modestly recommend being in mind the possibility of considering the use of no vertebrate animal models, as worms or insect as a first screening when it is reasonable. For the latter stages of the research we may to use other mammalian models. However, for certain lung diseases, conclusive points will arise more properly from the conjunction of one or more experimental studies carrying on in different species.

## AUTHOR CONTRIBUTIONS

Yamilé L. Hernández and Daniel Yero authored first draft of manuscript with academic input and expertise provided by Isidre Gibert and Juan M. Pinos-Rodríguez. All authors were involved in reviewing manuscript and have approved the final version.

## Conflict of Interest Statement

The authors declare that the research was conducted in the absence of any commercial or financial relationships that could be construed as a potential conflict of interest.

## References

[B1] Adamou J. E. Heinrichs J. H. Erwin A. L. Walsh W. Gayle T. Dormitzer M. (2001). Identification and characterization of a novel family of pneumococcal proteins that are protective against sepsis. *Infect. Immun.* 69 949–958 10.1128/IAI.69.2.949-958.2001 11159990 PMC97974

[B2] Aguilar D. Hanekom M. Mata D. Gey van Pittius N. C. van Helden P. D. Warren R. M. (2010). *Mycobacterium tuberculosis* strains with the Beijing genotype demonstrate variability in virulence associated with transmission. *Tuberculosis (Edinb.)* 90 319–325 10.1016/j.tube.2010.08.004 20832364

[B3] Aguilar D. Infante E. Martin C. Gormley E. Gicquel B. Hernandez Pando R. (2007). Immunological responses and protective immunity against tuberculosis conferred by vaccination of Balb/C mice with the attenuated *Mycobacterium tuberculosis* (phoP) SO2 strain. *Clin. Exp. Immunol.* 147 330–338 10.1111/j.1365-2249.2006.03284.x 17223975 PMC1810479

[B4] Alami S. Y. Kelly F. C. Race G. J. (1968). Pathogenicity of staphylococci: with special reference to the persitence of infection in mice. *Am. J. Pathol.* 53 577–589. 5677139 PMC2013414

[B5] Aldwell F. E. Brandt L. Fitzpatrick C. Orme I. M. (2005). Mice fed lipid-encapsulated *Mycobacterium bovis* BCG are protected against aerosol challenge with *Mycobacterium tuberculosis*. *Infect. Immun.* 73 1903–1905 10.1128/IAI.73.3.1903-1905.2005 15731098 PMC1064971

[B6] Alejo A. Tafalla C. (2011). Chemokines in teleost fish species. *Dev. Comp. Immunol.* 35 1215–1222 10.1016/j.dci.2011.03.011 21414348

[B7] Ali S. Champagne D. L. Spaink H. P. Richardson M. K. (2011). Zebrafish embryos and larvae: a new generation of disease models and drug screens. *Birth Defects Res. C Embryo Today* 93 115–133 10.1002/bdrc.20206 21671352

[B8] Alper S. Laws R. Lackford B. Boyd W. A. Dunlap P. Freedman J. H. (2008). Identification of innate immunity genes and pathways using a comparative genomics approach. *Proc. Natl. Acad. Sci. U.S.A.* 105 7016–7021 10.1073/pnas.0802405105 18463287 PMC2383967

[B9] Alper S. McElwee M. K. Apfeld J. Lackford B. Freedman J. H. Schwartz D. A. (2010). The *Caenorhabditis elegans* germ line regulates distinct signaling pathways to control lifespan and innate immunity. *J. Biol. Chem.* 285 1822–1828 10.1074/jbc.M109.057323 19923212 PMC2804340

[B10] Alvarez N. Otero O. Camacho F. Borrero R. Tirado Y. Puig A. (2013). Passive administration of purified secretory IgA from human colostrum induces protection against *Mycobacterium tuberculosis* in a murine model of progressive pulmonary infection. *BMC Immunol.* 14(Suppl. 1):S3 10.1186/1471-2172-14-S1-S3 PMC3582447 23458564

[B11] Anatoliĭ S. A. Antonovskaia I. I. Task S. I. Paderina E. M. (1971). [Comparative characteristics of some experimental models of staphylococcus infection]. *Zh. Mikrobiol. Epidemiol. Immunobiol.* 48 60–63. 5113376

[B12] Andreevskaia S. N. Chernousova L. N. Smirnova T. G. Larionova E. E. Kuz’min A. V. (2007). [Impact of *M. tuberculosis* genotype on survival in mice with experimental tuberculosis]. *Probl. Tuberk. Bolezn. Legk.* 7 45–50. 17718073

[B13] Ankeny R. A. (2001). The natural history of *Caenorhabditis elegans* research. *Nat. Rev. Genet.* 2 474–479 10.1038/35076538 11389464

[B14] Ansfield M. J. Woods D. E. Johanson W. G. Jr. (1977). Lung bacterial clearance in murine pneumococcal pneumonia. *Infect. Immun.* 17 195–204. 18404 10.1128/iai.17.1.195-204.1977 PMC421101

[B15] Apolónio J. Faleiro M. L. Miguel M. G. Neto L. (2014). No induction of antimicrobial resistance in *Staphylococcus aureus* and *Listeria monocytogenes* during continuous exposure to eugenol and citral. *FEMS Microbiol. Lett.* 354 92–101 10.1111/1574-6968.12440 24716611

[B16] Apt A. S. (2011). Are mouse models of human mycobacterial diseases relevant? Genetics says: “yes!” *Immunology* 134 109–115 10.1111/j.1365-2567.2011.03472.x 21896006 PMC3194219

[B17] Apt A. Kramnik I. (2009). Man and mouse TB: contradictions and solutions. *Tuberculosis (Edinb.)* 89 195–198 10.1016/j.tube.2009.02.002 19345146 PMC2705810

[B18] Aziz R. K. Kansal R. Abdeltawab N. F. Rowe S. L. Su Y. Carrigan D. (2007). Susceptibility to severe *Streptococcal sepsis*: use of a large set of isogenic mouse lines to study genetic and environmental factors. *Genes Immun.* 8 404–415 10.1038/sj.gene.6364402 17525705

[B19] Bakker-Woudenberg I. A. J. M. (2003). Experimental models of pulmonary infection. *J. Microbiol. Methods* 54 295–313 10.1016/S0167-7012(03)00118-0 12842477

[B20] Bakker-Woudenberg I. A. ten Kate M. T. Guo L. Working P. Mouton J. W. (2002). Ciprofloxacin in polyethylene glycol-coated liposomes: efficacy in rat models of acute or chronic *Pseudomonas aeruginosa* infection. *Antimicrob. Agents Chemother.* 46 2575–2581 10.1128/AAC.46.8.2575-2581.2002 12121935 PMC127349

[B21] Balloy V. Verma A. Kuravi S. Si-Tahar M. Chignard M. Ramphal R. (2007). The role of flagellin versus motility in acute lung disease caused by *Pseudomonas aeruginosa*. *J. Infect. Dis.* 196 289–296 10.1086/518610 17570117

[B22] Balls M. (2009). The origins and early days of the Three Rs concept. *Altern. Lab. Anim.* 37 255–265. 19678726 10.1177/026119290903700306

[B23] Balls M. Halder M. (2002). Progress in applying the three Rs of Russell & Burch to the testing of biological products. *Dev. Biol.* 111 3–13. 12678219

[B24] Barbier F. Andremont A. Wolff M. Bouadma L. (2013). Hospital-acquired pneumonia and ventilator-associated pneumonia: recent advances in epidemiology and management. *Curr. Opin. Pulm. Med.* 19 216–228 10.1097/MCP.0b013e32835f27be 23524477

[B25] Bartell P. F. Orr T. E. Geffen A. Iorio P. (1968). Experimental infection of mice with *Staphylococcus aureus*: evidence against alpha toxin and the terminal size of the bacterial population as determinants of lethality. *J. Infect. Dis.* 118 481–490 10.1093/infdis/118.5.481 4236131

[B26] Baumans V. (2004). Use of animals in experimental research: an ethical dilemma? *Gene Ther.* 11(Suppl. 1), S64–S66 10.1038/sj.gt.3302371 15454959

[B27] Baur S. Rautenberg M. Faulstich M. Grau T. Severin Y. Unger C. (2014). A nasal epithelial receptor for *Staphylococcus aureus* WTA governs adhesion to epithelial cells and modulates nasal colonization. *PLoS Pathog.* 10:e1004089 10.1371/journal.ppat.1004089 PMC4006915 24788600

[B28] Be N. A. Lamichhane G. Grosset J. Tyagi S. Cheng Q.-J. Kim K. S. (2008). Murine model to study the invasion and survival of *Mycobacterium tuberculosis* in the central nervous system. *J. Infect. Dis.* 198 1520–1528 10.1086/592447 18956986

[B29] Beale E. Li G. Tan M.-W. Rumbaugh K. P. (2006). *Caenorhabditis elegans* senses bacterial autoinducers. *Appl. Environ. Microbiol.* 72 5135–5137 10.1128/AEM.00611-06 16820523 PMC1489312

[B30] Beamer G. L. Turner J. (2005). Murine models of susceptibility to tuberculosis. *Arch. Immunol. Ther. Exp.* (*Warsz.*) 53 469–483. 16407780

[B31] Begun J. Gaiani J. M. Rohde H. Mack D. Calderwood S. B. Ausubel F. M. (2007). Staphylococcal biofilm exopolysaccharide protects against *Caenorhabditis elegans* immune defenses. *PLoS Pathog.* 3:e57 10.1371/journal.ppat.0030057 PMC1853117 17447841

[B32] Begun J. Sifri C. D. Goldman S. Calderwood S. B. Ausubel F. M. (2005). *Staphylococcus aureus* virulence factors identified by using a high-throughput *Caenorhabditis elegans*-killing model. *Infect. Immun.* 73 872–877 10.1128/IAI.73.2.872-877.2005 15664928 PMC547013

[B33] Bem R. A. Domachowske J. B. Rosenberg H. F. (2011). Animal models of human respiratory syncytial virus disease. *Am. J. Physiol. Lung Cell. Mol. Physiol.* 301 L148–L156 10.1152/ajplung.00065.2011 21571908 PMC3154630

[B34] Benyumov A. O. Hergert P. Herrera J. Peterson M. Henke C. Bitterman P. B. (2012). A novel zebrafish embryo xenotransplantation model to study primary human fibroblast motility in health and disease. *Zebrafish* 9 38–43 10.1089/zeb.2011.0705 22356695 PMC3308709

[B35] Berg R. D. Ramakrishnan L. (2012). Insights into tuberculosis from the zebrafish model. *Trends Mol. Med.* 18 689–690 10.1016/j.molmed.2012.10.002 23084762

[B36] Blair C. Naclerio R. M. Yu X. Thompson K. Sperling A. (2005). Role of type 1 T helper cells in the resolution of acute *Streptococcus pneumoniae* sinusitis: a mouse model. *J. Infect. Dis.* 192 1237–1244 10.1086/444544 16136467

[B37] Bloemendaal A. L. A. Vriens M. R. Jansen W. T. M. Borel Rinkes I. H. M. Verhoef J. Fluit A. C. (2011). Colonization and transmission of meticillin-susceptible and meticillin-resistant *Staphylococcus aureus* in a murine nasal colonization model. *J. Med. Microbiol.* 60 812–816 10.1099/jmm.0.027532-0 21317194

[B38] Boël G. Jin H. Pancholi V. (2005). Inhibition of cell surface export of group A streptococcal anchorless surface dehydrogenase affects bacterial adherence and antiphagocytic properties. *Infect. Immun.* 73 6237–6248 10.1128/IAI.73.10.6237-6248.2005 16177295 PMC1230963

[B39] Bogaert D. Hermans P. W. M. Adrian P. V. Rümke H. C. de Groot R. (2004). Pneumococcal vaccines: an update on current strategies. *Vaccine* 22 2209–2220 10.1016/j.vaccine.2003.11.038 15149779

[B40] Bolm M. Jansen W. T. M. Schnabel R. Chhatwal G. S. (2004). Hydrogen peroxide-mediated killing of *Caenorhabditis elegans*: a common feature of different streptococcal species. *Infect. Immun.* 72 1192–1194 10.1128/IAI.72.2.1192-1194.2004 14742574 PMC321644

[B41] Boman H. G. Nilsson I. Rasmuson B. (1972). Inducible antibacterial defence system in *Drosophila*. *Nature* 237 232–235 10.1038/237232a0 4625204

[B42] Borst L. B. Patterson S. K. Lanka S. Suyemoto M. M. Maddox C. W. (2013). Zebrafish (*Danio rerio*) as a screen for attenuation of Lancefield group C streptococci and a model for streptococcal pathogenesis. *Vet. Pathol.* 50 457–467 10.1177/0300985811424731 21997564

[B43] Bragonzi A. Copreni E. de Bentzmann S. Ulrich M. Conese M. (2004). Airway epithelial cell-pathogen interactions. *J. Cyst. Fibros.* 3(Suppl. 2), 197–201 10.1016/j.jcf.2004.05.041 15463958

[B44] Brannon M. K. Davis J. M. Mathias J. R. Hall C. J. Emerson J. C. Crosier P. S. (2009). *Pseudomonas aeruginosa* Type III secretion system interacts with phagocytes to modulate systemic infection of zebrafish embryos. *Cell. Microbiol.* 11 755–768 10.1111/j.1462-5822.2009.01288.x 19207728 PMC2933946

[B45] Briles D. E. Hollingshead S. K. Nabors G. S. Paton J. C. Brooks-Walter A. (2000). The potential for using protein vaccines to protect against otitis media caused by *Streptococcus pneumoniae*. *Vaccine* 19(Suppl. 1), S87–S95 10.1016/S0264-410X(00)00285-1 11163470

[B46] Brooke J. S. (2012). *Stenotrophomonas maltophilia*: an emerging global opportunistic pathogen. *Clin. Microbiol. Rev.* 25 2–41 10.1128/CMR.00019-11 22232370 PMC3255966

[B47] Cambier C. J. Takaki K. K. Larson R. P. Hernandez R. E. Tobin D. M. Urdahl K. B. (2014). Mycobacteria manipulate macrophage recruitment through coordinated use of membrane lipids. *Nature* 505 218–222 10.1038/nature12799 24336213 PMC3961847

[B48] Cantas L. Sørby J. R. T. Aleström P. Sørum H. (2012). Culturable gut microbiota diversity in zebrafish. *Zebrafish* 9 26–37 10.1089/zeb.2011.0712 22428747 PMC3308716

[B49] Cardona P.-J. Gordillo S. Díaz J. Tapia G. Amat I. Pallarés A. (2003). Widespread bronchogenic dissemination makes DBA/2 mice more susceptible than C57BL/6 mice to experimental aerosol infection with *Mycobacterium tuberculosis*. *Infect. Immun.* 71 5845–5854 10.1128/IAI.71.10.5845-5854.2003 14500506 PMC201050

[B50] Castillo-Rodal A. I. Castañón-Arreola M. Hernández-Pando R. Calva J. J. Sada-Díaz E. López-Vidal Y. (2006). *Mycobacterium bovis* BCG substrains confer different levels of protection against *Mycobacterium tuberculosis* infection in a BALB/c model of progressive pulmonary tuberculosis. *Infect. Immun.* 74 1718–1724 10.1128/IAI.74.3.1718-1724.2006 16495544 PMC1418655

[B51] Castonguay-Vanier J. Vial L. Tremblay J. Déziel E. (2010). *Drosophila melanogaster* as a model host for the *Burkholderia cepacia* complex. *PLoS ONE* 5:e11467 10.1371/journal.pone.0011467 PMC2902503 20635002

[B52] Cezairliyan B. Vinayavekhin N. Grenfell-Lee D. Yuen G. J. Saghatelian A. Ausubel F. M. (2013). Identification of *Pseudomonas aeruginosa* phenazines that kill *Caenorhabditis elegans*. *PLoS Pathog.* 9:e1003101 10.1371/journal.ppat.1003101 PMC3536714 23300454

[B53] Chen B. Weisbrod T. R. Hsu T. Sambandamurthy V. Vieira-Cruz D. Chibbaro A. (2011). Einstein Contained Aerosol Pulmonizer (ECAP): improved biosafety for multi-drug resistant (MDR) and extensively drug resistant (XDR) *Mycobacterium tuberculosis* aerosol infection studies. *Appl. Biosaf.* 16 134–138. 23413363 10.1177/153567601101600302 PMC3569622

[B54] Chmiel J. F. Konstan M. W. Knesebeck J. E. Hilliard J. B. Bonfield T. L. Dawson D. V. (1999). IL-10 attenuates excessive inflammation in chronic *Pseudomonas* infection in mice. *Am. J. Respir. Crit. Care Med.* 160 2040–2047 10.1164/ajrccm.160.6.9901043 10588626

[B55] Chun J. Prince A. (2009). Ca^2+^ signaling in airway epithelial cells facilitates leukocyte recruitment and transepithelial migration. *J. Leukoc. Biol.* 86 1135–1144 10.1189/jlb.0209072 19605699 PMC3192023

[B56] Clatworthy A. E. Lee J. S.-W. Leibman M. Kostun Z. Davidson A. J. Hung D. T. (2009). *Pseudomonas aeruginosa* infection of zebrafish involves both host and pathogen determinants. *Infect. Immun.* 77 1293–1303 10.1128/IAI.01181-08 19168742 PMC2663173

[B57] Collie D. Govan J. Wright S. Thornton E. Tennant P. Smith S. (2013). A lung segmental model of chronic *Pseudomonas* infection in sheep. *PLoS ONE* 8:e67677 10.1371/journal.pone.0067677 PMC3706528 23874438

[B58] Collins J. F. Anzueto A. A. Peters J. I. de los Santos R. Gonzalez D. C. Johanson W. G. (1991). Elastase activity in bronchoalveolar lavage fluid from oxygen-exposed, *Pseudomonas*-infected baboons. *Lung* 169 165–179 10.1007/BF02714152 1895779

[B59] Cooper A. M. (2014). Mouse model of tuberculosis. *Cold Spring Harb. Perspect. Med.* 10.1101/cshperspect.a018556 [Epub ahead of print]. PMC4315921 25256174

[B60] Cosma C. L. Klein K. Kim R. Beery D. Ramakrishnan L. (2006a). *Mycobacterium marinum* Erp is a virulence determinant required for cell wall integrity and intracellular survival. *Infect. Immun.* 74 3125–3133 10.1128/IAI.02061-05 16714540 PMC1479242

[B61] Cosma C. L. Swaim L. E. Volkman H. Ramakrishnan L. Davis J. M. (2006b). Zebrafish and frog models of *Mycobacterium marinum* infection. *Curr. Protoc. Microbiol. Chap.* 10 Unit 10B.2. 10.1002/0471729256.mc10b02s3 18770575

[B62] Couillault C. Ewbank J. J. (2002). Diverse bacteria are pathogens of *Caenorhabditis elegans*. *Infect. Immun.* 70 4705–4707 10.1128/IAI.70.8.4705-4707.2002 12117988 PMC128124

[B63] Coutinho H. D. M. Falcao-Silva V. S. Goncalves G. F. (2008). Pulmonary bacterial pathogens in cystic fibrosis patients and antibiotic therapy: a tool for the health workers. *Int. Arch. Med.* 1 24 10.1186/1755-7682-1-24 PMC2586015 18992146

[B64] Cressey D. (2014). Fish-kill method questioned. *Nature* 506 419–420 10.1038/506419a 24572404

[B65] Cronan M. R. Tobin D. M. (2014). Fit for consumption: zebrafish as a model for tuberculosis. *Dis. Model. Mech.* 7 777–784 10.1242/dmm.016089 24973748 PMC4073268

[B66] Cui C. Benard E. L. Kanwal Z. Stockhammer O. W. van der Vaart M. Zakrzewska A. (2011). Infectious disease modeling and innate immune function in zebrafish embryos. *Methods Cell Biol.* 105 273–308 10.1016/B978-0-12-381320-6.00012-6 21951535

[B67] Damy S. B. Camargo R. S. Chammas R. Figueiredo L. F. (2010). [Fundamental aspects on animal research as applied to experimental surgery]. *Rev. Assoc. Med. Bras.* 1992 56 103–111 10.1590/S0104-42302010000100024 20339795

[B68] Darby C. (2005). “Interactions with microbial pathogens,” in *WormBook*, ed. The *C. elegans* Research Community, WormBook 10.1895/wormbook.1.21.1 PMC4781414 18050390

[B69] D’Argenio D. A. Gallagher L. A. Berg C. A. Manoil C. (2001). *Drosophila* as a model host for *Pseudomonas aeruginosa* infection. *J. Bacteriol.* 183 1466–1471 10.1128/JB.183.4.1466-1471.2001 11157963 PMC95024

[B70] Davis J. M. Clay H. Lewis J. L. Ghori N. Herbomel P. Ramakrishnan L. (2002). Real-time visualization of mycobacterium-macrophage interactions leading to initiation of granuloma formation in zebrafish embryos. *Immunity* 17 693–702 10.1016/S1074-7613(02)00475-2 12479816

[B71] Davis J. M. Ramakrishnan L. (2009). The role of the granuloma in expansion and dissemination of early tuberculous infection. *Cell* 136 37–49 10.1016/j.cell.2008.11.014 19135887 PMC3134310

[B72] Dean S. N. Bishop B. M. van Hoek M. L. (2011). Susceptibility of *Pseudomonas aeruginosa* Biofilm to Alpha-Helical Peptides: D-enantiomer of LL-37. *Front. Microbiol.* 2:128 10.3389/fmicb.2011.00128 PMC3131519 21772832

[B73] Denny P. (2005). Editorial. Mouse models of infectious disease. *Brief. Funct. Genomic. Proteomic.* 4 201–202 10.1093/bfgp/4.3.201 16420745

[B74] Desbois A. P. Coote P. J. (2011). Wax moth larva (*Galleria mellonella*): an in vivo model for assessing the efficacy of antistaphylococcal agents. *J. Antimicrob. Chemother.* 66 1785–1790 10.1093/jac/dkr198 21622972

[B75] De Steenwinkel J. E. M. De Knegt G. J. Ten Kate M. T. Van Belkum A. Verbrugh H. A. Hernandez-Pando R. (2009). Immunological parameters to define infection progression and therapy response in a well-defined tuberculosis model in mice. *Int. J. Immunopathol. Pharmacol.* 22 723–734. 19822089 10.1177/039463200902200318

[B76] Dharmadhikari A. S. Nardell E. A. (2008). What animal models teach humans about tuberculosis. *Am. J. Respir. Cell Mol. Biol.* 39 503–508 10.1165/rcmb.2008-0154TR 18556589 PMC4674828

[B77] Di Bonaventura G. Pompilio A. Zappacosta R. Petrucci F. Fiscarelli E. Rossi C. (2010). Role of excessive inflammatory response to *Stenotrophomonas maltophilia* lung infection in DBA/2 mice and implications for cystic fibrosis. *Infect. Immun.* 78 2466–2476 10.1128/IAI.01391-09 20308302 PMC2876550

[B78] Dionne M. S. Ghori N. Schneider D. S. (2003). *Drosophila melanogaster* is a genetically tractable model host for *Mycobacterium marinum*. *Infect. Immun.* 71 3540–3550 10.1128/IAI.71.6.3540-3550.2003 12761139 PMC155752

[B79] Di Pietrantonio T. Schurr E. (2005). Mouse models for the genetic study of tuberculosis susceptibility. *Brief. Funct. Genomic. Proteomic.* 4 277–292 10.1093/bfgp/4.3.277 16420753

[B80] Dunbar T. L. Yan Z. Balla K. M. Smelkinson M. G. Troemel E. R. (2012). *C. elegans* detects pathogen-induced translational inhibition to activate immune signaling. *Cell Host Microbe* 11, 375–386 10.1016/j.chom.2012.02.008 22520465 PMC3334869

[B81] Endo Y. Takahashi M. Iwaki D. Ishida Y. Nakazawa N. Kodama T. (2012). Mice deficient in ficolin, a lectin complement pathway recognition molecule, are susceptible to *Streptococcus pneumoniae* infection. *J. Immunol.* 189 5860–5866 10.4049/jimmunol.1200836 23150716

[B82] Erdem H. Inan A. Altındis S. Carevic B. Askarian M. Cottle L. (2014). Surveillance, control and management of infections in intensive care units in Southern Europe, Turkey and Iran – a prospective multicenter point prevalence study. *J. Infect.* 68 131–140 10.1016/j.jinf.2013.11.001 24269951

[B83] Evans B. A. Rozen D. E. (2012). A *Streptococcus pneumoniae* infection model in larvae of the wax moth *Galleria mellonella*. *Eur. J. Clin. Microbiol. Infect. Dis.* 31 2653–2660 10.1007/s10096-012-1609-7 22466968

[B84] Evans S. E. Tuvim M. J. Zhang J. Larson D. T. García C. D. Martinez-Pro S. (2010). Host lung gene expression patterns predict infectious etiology in a mouse model of pneumonia. *Respir. Res.* 11:101 10.1186/1465-9921-11-101 PMC2914038 20653947

[B85] Feinbaum R. L. Urbach J. M. Liberati N. T. Djonovic S. Adonizio A. Carvunis A.-R. (2012). Genome-Wide Identification of *Pseudomonas aeruginosa* virulence-related genes using a *Caenorhabditis elegans* infection model. *PLoS Pathog.* 8:e1002813 10.1371/journal.ppat.1002813 PMC3406104 22911607

[B86] Fernandes C. A. Vanbever R. (2009). Preclinical models for pulmonary drug delivery. *Expert Opin. Drug Deliv.* 6 1231–1245 10.1517/17425240903241788 19852680

[B87] Fernandez S. Cisney E. D. Hall S. I. Ulrich R. G. (2011). Nasal immunity to staphylococcal toxic shock is controlled by the nasopharynx-associated lymphoid tissue. *Clin. Vaccine Immunol.* 18 667–675 10.1128/CVI.00477-10 21325486 PMC3122560

[B88] Ferrer-Navarro M. Planell R. Yero D. Mongiardini E. Torrent G. Huedo P. (2013). Abundance of the Quorum-Sensing factor Ax21 in four strains of *Stenotrophomonas maltophilia* correlates with mortality rate in a New Zebrafish model of infection. *PLoS ONE* 8:e67207 10.1371/journal.pone.0067207 PMC3693955 23840626

[B89] Fuchs H. Gailus-Durner V. Adler T. Pimentel J. A. A. Becker L. Bolle I. (2009). The German mouse clinic: a platform for systemic phenotype analysis of mouse models. *Curr. Pharm. Biotechnol.* 10 236–243 10.2174/138920109787315051 19199957

[B90] Gan Y.-H. Chua K. L. Chua H. H. Liu B. Hii C. S. Chong H. L. (2002). Characterization of *Burkholderia pseudomallei* infection and identification of novel virulence factors using a *Caenorhabditis elegans* host system. *Mol. Microbiol.* 44 1185–1197 10.1046/j.1365-2958.2002.02957.x 12068805

[B91] Gao W. Chua K. Davies J. K. Newton H. J. Seemann T. Harrison P. F. (2010). Two novel point mutations in clinical *Staphylococcus aureus* reduce linezolid susceptibility and switch on the stringent response to promote persistent infection. *PLoS Pathog.* 6:e1000944 10.1371/journal.ppat.1000944 PMC2883592 20548948

[B92] Garsin D. A. Sifri C. D. Mylonakis E. Qin X. Singh K. V. Murray B. E. (2001). A simple model host for identifying Gram-positive virulence factors. *Proc. Natl. Acad. Sci. U.S.A.* 98 10892–10897 10.1073/pnas.191378698 11535834 PMC58570

[B93] Garsin D. A. Villanueva J. M. Begun J. Kim D. H. Sifri C. D. Calderwood S. B. (2003). Long-lived *C. elegans* daf-2 mutants are resistant to bacterial pathogens. *Science* 300:1921 10.1126/science.1080147 12817143

[B94] Geng Y. Wang K. Chen D. Huang X. He M. Yin Z. (2010). *Stenotrophomonas maltophilia*, an emerging opportunist pathogen for cultured channel catfish, *Ictalurus punctatus*, in China. *Aquaculture* 308 132–135 10.1016/j.aquaculture.2010.08.032

[B95] George S. E. Kohan M. J. Whitehouse D. A. Creason J. P. Kawanishi C. Y. Sherwood R. L. (1991). Distribution, clearance, and mortality of environmental pseudomonads in mice upon intranasal exposure. *Appl. Environ. Microbiol.* 57 2420–2425. 1722663 10.1128/aem.57.8.2420-2425.1991 PMC183589

[B96] Gerber J. Raivich G. Wellmer A. Noeske C. Kunst T. Werner A. (2001). A mouse model of *Streptococcus pneumoniae* meningitis mimicking several features of human disease. *Acta Neuropathol. (Berl.)* 101 499–508. 11484822 10.1007/s004010000326

[B97] Gibreel T. M. Upton M. (2013). Synthetic epidermicin NI01 can protect *Galleria mellonella* larvae from infection with *Staphylococcus aureus.* *J. Antimicrob. Chemother.* 68 2269–2273 10.1093/jac/dkt195 23711896

[B98] Glavis-Bloom J. Muhammed M. Mylonakis E. (2012). Of model hosts and man: using *Caenorhabditis elegans*, *Drosophila melanogaster* and *Galleria mellonella* as model hosts for infectious disease research. *Adv. Exp. Med. Biol.* 710 11–17 10.1007/978-1-4419-5638-5_2 22127881

[B99] Gosselin D. DeSanctis J. Boulé M. Skamene E. Matouk C. Radzioch D. (1995). Role of tumor necrosis factor alpha in innate resistance to mouse pulmonary infection with *Pseudomonas aeruginosa*. *Infect. Immun.* 63 3272–3278 10.1007/978-1-4419-5638-5_2 7642255 PMC173451

[B100] Gosselin D. Stevenson M. M. Cowley E. A. Griesenbach U. Eidelman D. H. Boulé M. (1998). Impaired ability of Cftr knockout mice to control lung infection with *Pseudomonas aeruginosa*. *Am. J. Respir. Crit. Care Med.* 157 1253–1262 10.1164/ajrccm.157.4.9702081 9563748

[B101] Green R. M. Gally F. Keeney J. G. Alper S. Gao B. Han M. (2009). Impact of cigarette smoke exposure on innate immunity: a *Caenorhabditis elegans* model. *PLoS ONE* 4:e6860 10.1371/journal.pone.0006860 PMC2729919 19718433

[B102] Grumbach F. Canetti G. Grosset J. le Lirzin M. (1967). Late results of long-term intermittent chemotherapy of advanced, murine tuberculosis: limits of the murine model. *Tubercle* 48 11–26 10.1016/S0041-3879(67)80047-3 6034625

[B103] Hall A. E. Patel P. R. Domanski P. J. Prater B. D. Gorovits E. L. Syribeys P. J. (2007). A panel of monoclonal antibodies recognizing the *Staphylococcus epidermidis* fibrinogen-binding MSCRAMM SdrG. *Hybridoma* 26 28–34 10.1089/hyb.2006.039 17316083

[B104] Hammarén M. M. Oksanen K. E. Nisula H. M. Luukinen B. V. Pesu M. Rämet M. (2014). Adequate Th2-type response associates with restricted bacterial growth in latent mycobacterial infection of zebrafish. *PLoS Pathog.* 10:e1004190 10.1371/journal.ppat.1004190 PMC4072801 24968056

[B105] Hart D. A. Green F. Whidden P. Henkin J. Woods D. E. (1993). Exogenous rh-urokinase modifies inflammation and *Pseudomonas aeruginosa* infection in a rat chronic pulmonary infection model. *Can. J. Microbiol.* 39 1127–1134 10.1139/m93-170 8131109

[B106] Hendriksen C. F. M. (2002). Refinement, reduction, and replacement of animal use for regulatory testing: current best scientific practices for the evaluation of safety and potency of biologicals. *ILAR J.* 43(Suppl.), S43–S48. 12388851 10.1093/ilar.43.suppl_1.s43

[B107] Hernandez-Pando R. de la Luz Streber M. Orozco H. Arriaga K. Pavon L. Marti O. (1998). Emergent immunoregulatory properties of combined glucocorticoid and anti-glucocorticoid steroids in a model of tuberculosis. *QJM* 91 755–766 10.1093/qjmed/91.11.755 10024939

[B108] Hernández-Pando R. Orozcoe H. Sampieri A. Pavón L. Velasquillo C. Larriva-Sahd J. (1996). Correlation between the kinetics of Th1, Th2 cells and pathology in a murine model of experimental pulmonary tuberculosis. *Immunology* 89 26–33. 8911136 PMC1456655

[B109] Holland M. C. H. Lambris J. D. (2004). A functional C5a anaphylatoxin receptor in a teleost species. *J. Immunol.* 172 349–355 10.4049/jimmunol.172.1.349 14688343

[B110] Hu D.-L. Omoe K. Narita K. Cui J.-C. Shinagawa K. Nakane A. (2006). Intranasal vaccination with a double mutant of staphylococcal enterotoxin C provides protection against *Staphylococcus aureus* infection. *Microbes Infect.* 8 2841–2848 10.1016/j.micinf.2006.09.001 17090392

[B111] Huedo P. Yero D. Martínez-Servat S. Estibariz I. Planell R. Martínez P. (2014). Two different rpf clusters distributed among a population of *Stenotrophomonas maltophilia* clinical strains display differential diffusible signal factor production and virulence regulation. *J. Bacteriol.* 196 2431–2442 10.1128/JB.01540-14 24769700 PMC4054175

[B112] Hulme S. E. Whitesides G. M. (2011). Chemistry and the worm: *Caenorhabditis elegans* as a platform for integrating chemical and biological research. *Angew. Chem. Int. Ed. Engl.* 50 4774–4807 10.1002/anie.201005461 21500322

[B113] Hunter R. L. Jagannath C. Actor J. K. (2007). Pathology of postprimary tuberculosis in humans and mice: contradiction of long-held beliefs. *Tuberculosis (Edinb.)* 87 267–278 10.1016/j.tube.2006.11.003 17369095

[B114] Huzella L. M. Buckley M. J. Alves D. A. Stiles B. G. Krakauer T. (2009). Central roles for IL-2 and MCP-1 following intranasal exposure to SEB: a new mouse model. *Res. Vet. Sci.* 86 241–247 10.1016/j.rvsc.2008.07.020 18793785

[B115] Hyde D. M. Hamid Q. Irvin C. G. (2009). Anatomy, pathology, and physiology of the tracheobronchial tree: emphasis on the distal airways. *J. Allergy Clin. Immunol.* 124 S72–S77 10.1016/j.jaci.2009.08.048 19962039

[B116] Igboin C. O. Griffen A. L. Leys E. J. (2012). The *Drosophila melanogaster* host model. *J. Oral Microbiol.* 4:10368 10.3402/jom.v4i0.10368 PMC3285217 22368770

[B117] Irvin C. G. Bates J. H. T. (2003). Measuring the lung function in the mouse: the challenge of size. *Respir. Res.* 4:4 10.1186/rr199 PMC184039 12783622

[B118] Iwata M. Sato A. (1991). Morphological and immunohistochemical studies of the lungs and bronchus-associated lymphoid tissue in a rat model of chronic pulmonary infection with *Pseudomonas aeruginosa*. *Infect. Immun.* 59 1514–1520. 2004830 10.1128/iai.59.4.1514-1520.1991 PMC257870

[B119] Jain M. Bar-Meir M. McColley S. Cullina J. Potter E. Powers C. (2008). Evolution of *Pseudomonas aeruginosa* type III secretion in cystic fibrosis: a paradigm of chronic infection. *Transl. Res. J. Lab. Clin. Med.* 152 257–264 10.1016/j.trsl.2008.10.003 PMC2628760 19059160

[B120] Jakab G. J. (1976). Factors influencing the immune enhancement of intrapulmonary bactericidal mechanisms. *Infect. Immun.* 14 389–398. 9364 10.1128/iai.14.2.389-398.1976 PMC420896

[B121] Jander G. Rahme L. G. Ausubel F. M. (2000). Positive correlation between virulence of *Pseudomonas aeruginosa* mutants in mice and insects. *J. Bacteriol.* 182 3843–3845 10.1128/JB.182.13.3843-3845.2000 10851003 PMC94559

[B122] Jansen W. T. M. Bolm M. Balling R. Chhatwal G. S. Schnabel R. (2002). Hydrogen peroxide-mediated killing of *Caenorhabditis elegans* by *Streptococcus pyogenes*. *Infect. Immun.* 70 5202–5207 10.1128/IAI.70.9.5202-5207.2002 12183571 PMC128270

[B123] JebaMercy G. Pandian S. K. Balamurugan K. (2011). Changes in *Caenorhabditis elegans* life span and selective innate immune genes during *Staphylococcus aureus* infection. *Folia Microbiol. (Praha)* 56 373–380 10.1007/s12223-011-0060-y 21853381

[B124] Jones R. N. (2010). Microbial etiologies of hospital-acquired bacterial pneumonia and ventilator-associated bacterial pneumonia. *Clin. Infect. Dis.* 51(Suppl. 1), S81–S87 10.1086/653053 20597676

[B125] Kadioglu A. Andrew P. W. (2004). The innate immune response to pneumococcal lung infection: the untold story. *Trends Immunol.* 25 143–149 10.1016/j.it.2003.12.006 15036042

[B126] Kanther M. Rawls J. F. (2010). Host-microbe interactions in the developing zebrafish. *Curr. Opin. Immunol.* 22 10–19 10.1016/j.coi.2010.01.006 20153622 PMC3030977

[B127] Karakousis P. C. Yoshimatsu T. Lamichhane G. Woolwine S. C. Nuermberger E. L. Grosset J. (2004). Dormancy phenotype displayed by extracellular *Mycobacterium tuberculosis* within artificial granulomas in mice. *J. Exp. Med.* 200 647–657 10.1084/jem.20040646 15353557 PMC2212740

[B128] Kato J. Kim H.-E. Takiguchi N. Kuroda A. Ohtake H. (2008). *Pseudomonas aeruginosa* as a model microorganism for investigation of chemotactic behaviors in ecosystem. *J. Biosci. Bioeng.* 106 1–7 10.1263/jbb.106.1 18691523

[B129] Kaushal D. Mehra S. Didier P. J. Lackner A. A. (2012). The non-human primate model of tuberculosis. *J. Med. Primatol.* 41 191–201 10.1111/j.1600-0684.2012.00536.x 22429048 PMC3961469

[B130] Kelly B. P. Furney S. K. Jessen M. T. Orme I. M. (1996). Low-dose aerosol infection model for testing drugs for efficacy against *Mycobacterium tuberculosis*. *Antimicrob. Agents Chemother.* 40 2809–2812. 9124846 10.1128/aac.40.12.2809 PMC163627

[B131] Kim D. H. Feinbaum R. Alloing G. Emerson F. E. Garsin D. A. Inoue H. (2002). A conserved p38 MAP kinase pathway in *Caenorhabditis elegans* innate immunity. *Science* 297 623–626 10.1126/science.1073759 12142542

[B132] Kubica M. Guzik K. Koziel J. Zarebski M. Richter W. Gajkowska B. (2008). A potential new pathway for *Staphylococcus aureus* dissemination: the silent survival of *S. aureus* phagocytosed by human monocyte-derived macrophages. *PLoS ONE* 3:e1409 10.1371/journal.pone.0001409 PMC2169301 18183290

[B133] Kurahashi K. Sawa T. Ota M. Kajikawa O. Hong K. Martin T. R. (2009). Depletion of phagocytes in the reticuloendothelial system causes increased inflammation and mortality in rabbits with *Pseudomonas aeruginosa* pneumonia. *Am. J. Physiol. Lung Cell. Mol. Physiol.* 296 L198–L209 10.1152/ajplung.90472.2008 19028978 PMC2643994

[B134] Kurz C. L. Ewbank J. J.(2000). *Caenorhabditis elegans* for the study of host-pathogen interactions. *Trends Microbiol.* 8 142–144 10.1016/S0966-842X(99)01691-1 10707068

[B135] Kurz C. L. Ewbank J. J. (2007). Infection in a dish: high-throughput analyses of bacterial pathogenesis. *Curr. Opin. Microbiol.* 10 10–16 10.1016/j.mib.2006.12.001 17178462

[B136] Lebeaux D. Chauhan A. Rendueles O. Beloin C. (2013). From in vitro to in vivo models of bacterial Biofilm-related infections. *Pathogens* 2 288–356 10.3390/pathogens2020288 25437038 PMC4235718

[B137] Ledizet M. Murray T. S. Puttagunta S. Slade M. D. Quagliarello V. J. Kazmierczak B. I. (2012). The ability of virulence factor expression by *Pseudomonas aeruginosa* to predict clinical disease in hospitalized patients. *PLoS ONE* 7:e49578 10.1371/journal.pone.0049578 PMC3495863 23152923

[B138] Lee S.-H. Wong R.-R. Chin C.-Y. Lim T.-Y. Eng S.-A. Kong C. (2013). *Burkholderia pseudomallei* suppresses *Caenorhabditis elegans* immunity by specific degradation of a GATA transcription factor. *Proc. Natl. Acad. Sci. U.S.A.* 110 15067–15072 10.1073/pnas.1311725110 23980181 PMC3773767

[B139] Lemaitre B. Hoffmann J. (2007). The host defense of *Drosophila melanogaster*. *Annu. Rev. Immunol.* 25 697–743 10.1146/annurev.immunol.25.022106.141615 17201680

[B140] Letamendia A. Quevedo C. Ibarbia I. Virto J. M. Holgado O. Diez M. (2012). Development and validation of an automated high-throughput system for zebrafish in vivo screenings. *PLoS ONE* 7:e36690 10.1371/journal.pone.0036690 PMC3352927 22615792

[B141] Leung C. Chijioke O. Gujer C. Chatterjee B. Antsiferova O. Landtwing V. (2013). Infectious diseases in humanized mice. *Eur. J. Immunol.* 43 2246–2254 10.1002/eji.201343815 23913412

[B142] Li Y. Hu B. (2012). Establishment of multi-site infection model in zebrafish larvae for studying *Staphylococcus aureus* infectious disease. *J. Genet. Genomics* 39 521–534 10.1016/j.jgg.2012.07.006 23021551

[B143] Limmer S. Quintin J. Hetru C. Ferrandon D. (2011). Virulence on the fly: *Drosophila melanogaster* as a model genetic organism to decipher host-pathogen interactions. *Curr. Drug Targets* 12 978–999 10.2174/138945011795677818 21366519

[B144] Lin B. Chen S. Cao Z. Lin Y. Mo D. Zhang H. (2007). Acute phase response in zebrafish upon *Aeromonas salmonicida* and *Staphylococcus aureus* infection: striking similarities and obvious differences with mammals. *Mol. Immunol.* 44 295–301 10.1016/j.molimm.2006.03.001 16630661

[B145] Lipscomb M. F. Hutt J. Lovchik J. Wu T. Lyons C. R. (2010). The pathogenesis of acute pulmonary viral and bacterial infections: investigations in animal models. *Annu. Rev. Pathol.* 5 223–252 10.1146/annurev-pathol-121808-102153 19824827

[B146] López Y. Falero-Díaz G. Yero D. Solís R. L. Sarmiento M. E. Acosta A. (2010). Antibodies in the protection against mycobacterial infections: what have we learned? *Procedia Vaccinol.* 2 172–177 10.1016/j.provac.2010.07.011

[B147] López Y. Yero D. Falero-Diaz G. Olivares N. Sarmiento M. E. Sifontes S. (2009). Induction of a protective response with an IgA monoclonal antibody against *Mycobacterium tuberculosis* 16kDa protein in a model of progressive pulmonary infection. *Int. J. Med. Microbiol.* 299 447–452 10.1016/j.ijmm.2008.10.007 19157977

[B148] López Y. Yero D. Rodríguez S. S. Infante Bourzac J. F. Sarmiento M. E. Arzuaga N. O. (2006). Immunization of mice with a *Mycobacterium tuberculosis* genomic expression library results in lower bacterial load in lungs after challenge with BCG. *Tuberculosis (Edinb.)* 86 247–254 10.1016/j.tube.2006.01.004 16647298

[B149] Lü A.-J. Hu X.-C. Wang Y. Ming Q.-L. Hu H.-X. (2012). Immune response in the skin of zebrafish, *Danio rerio* (Hamilton), against *Staphylococcus* chromogenes. *J. Fish Dis.* 35 699–704 10.1111/j.1365-2761.2012.01371.x 22537075

[B150] Lugo-Villarino G. Balla K. M. Stachura D. L. Bañuelos K. Werneck M. B. F. Traver D. (2010). Identification of dendritic antigen-presenting cells in the zebrafish. *Proc. Natl. Acad. Sci. U.S.A.* 107 15850–15855 10.1073/pnas.1000494107 20733076 PMC2936643

[B151] Luna C. M. Baquero S. Gando S. Patrón J. R. Morato J. G. Sibila O. (2007). Experimental severe *Pseudomonas aeruginosa* pneumonia and antibiotic therapy in piglets receiving mechanical ventilation. *Chest* 132 523–531 10.1378/chest.07-0185 17699131

[B152] Luna C. M. Sibila O. Agusti C. Torres A. (2009). Animal models of ventilator-associated pneumonia. *Eur. Respir. J.* 33 182–188 10.1183/09031936.00046308 19118229

[B153] Lyons C. R. Lovchik J. Hutt J. Lipscomb M. F. Wang E. Heninger S. (2004). Murine model of pulmonary anthrax: kinetics of dissemination, histopathology, and mouse strain susceptibility. *Infect. Immun.* 72 4801–4809 10.1128/IAI.72.8.4801-4809.2004 15271942 PMC470666

[B154] Mall M. A. (2008). Role of cilia, mucus, and airway surface liquid in mucociliary dysfunction: lessons from mouse models. *J. Aerosol Med. Pulm. Drug Deliv.* 21 13–24 10.1089/jamp.2007.0659 18518828

[B155] Mall M. Grubb B. R. Harkema J. R. O’Neal W. K. Boucher R. C. (2004). Increased airway epithelial Na^+^ absorption produces cystic fibrosis-like lung disease in mice. *Nat. Med.* 10 487–493 10.1038/nm1028 15077107

[B156] Martin J. S. Renshaw S. A. (2009). Using in vivo zebrafish models to understand the biochemical basis of neutrophilic respiratory disease. *Biochem. Soc. Trans.* 37 830–837 10.1042/BST0370830 19614603

[B157] Mason C. M. Kolls J. K. Nelson S. (1995). Pathogenesis and host defense in pulmonary infections. *Curr. Opin. Pulm. Med.* 1 163–170. 9363048

[B158] McKay G. A. Woods D. E. MacDonald K. L. Poole K. (2003). Role of phosphoglucomutase of *Stenotrophomonas maltophilia* in lipopolysaccharide biosynthesis, virulence, and antibiotic resistance. *Infect. Immun.* 71 3068–3075 10.1128/IAI.71.6.3068-3075.2003 12761084 PMC155759

[B159] McMorran B. J. Palmer J. S. Lunn D. P. Oceandy D. Costelloe E. O. Thomas G. R. (2001). G551D CF mice display an abnormal host response and have impaired clearance of *Pseudomonas* lung disease. *Am. J. Physiol. Lung Cell. Mol. Physiol.* 281 L740–L747. 11504703 10.1152/ajplung.2001.281.3.L740

[B160] Means T. K. Aballay A. (2011). Models to study ancient host-pathogen interactions: lessons from Crete. *EMBO Rep.* 12 5–7 10.1038/embor.2010.205 21164515 PMC3024139

[B161] Meeker N. D. Trede N. S. (2008). Immunology and zebrafish: spawning new models of human disease. *Dev. Comp. Immunol.* 32 745–757 10.1016/j.dci.2007.11.011 18222541

[B162] Meijer A. H. Spaink H. P. (2011). Host-pathogen interactions made transparent with the zebrafish model. *Curr. Drug Targets* 12 1000–1017 10.2174/138945011795677809 21366518 PMC3319919

[B163] Mitra S. Alnabulsi A. Secombes C. J. Bird S. (2010). Identification and characterization of the transcription factors involved in T-cell development, t-bet, stat6 and foxp3, within the zebrafish, *Danio rerio*. *FEBS J.* 277 128–147 10.1111/j.1742-4658.2009.07460.x 19961539

[B164] Mizgerd J. P. Skerrett S. J. (2008). Animal models of human pneumonia. *Am. J. Physiol. Lung Cell. Mol. Physiol.* 294 L387–L398 10.1152/ajplung.00330.2007 18162603

[B165] Mollenkopf H.-J. Kursar M. Kaufmann S. H. E. (2004). Immune response to postprimary tuberculosis in mice: *Mycobacterium tuberculosis* and *Mycobacterium bovis* bacille Calmette-Guérin induce equal protection. *J. Infect. Dis.* 190 588–597 10.1086/422394. 15243936

[B166] Moreau-Marquis S. Stanton B. A. O’Toole G. A. (2008). *Pseudomonas aeruginosa* biofilm formation in the cystic fibrosis airway. *Pulm. Pharmacol. Ther.* 21 595–599 10.1016/j.pupt.2007.12.001 18234534 PMC2542406

[B167] Morissette C. Skamene E. Gervais F. (1995). Endobronchial inflammation following *Pseudomonas aeruginosa* infection in resistant and susceptible strains of mice. *Infect. Immun.* 63 1718–1724. 7729877 10.1128/iai.63.5.1718-1724.1995 PMC173215

[B168] Morsczeck C. Prokhorova T. Sigh J. Pfeiffer M. Bille-Nielsen M. Petersen J. (2008). *Streptococcus pneumoniae*: proteomics of surface proteins for vaccine development. *Clin. Microbiol. Infect.* 14 74–81 10.1111/j.1469-0691.2007.01878.x 18034862

[B169] Moser C. Jensen P. O. Kobayashi O. Hougen H. P. Song Z. Rygaard J. (2002). Improved outcome of chronic *Pseudomonas aeruginosa* lung infection is associated with induction of a Th1-dominated cytokine response. *Clin. Exp. Immunol.* 127 206–213 10.1046/j.1365-2249.2002.01731.x 11876741 PMC1906339

[B170] Moser C. Johansen H. K. Song Z. Hougen H. P. Rygaard J. Høiby N. (1997). Chronic *Pseudomonas aeruginosa* lung infection is more severe in Th2 responding BALB/c mice compared to Th1 responding C3H/HeN mice. *Acta Pathol. Microbiol. Immunol. Scand.* 105 838–842 10.1111/j.1699-0463.1997.tb05092.x 9393554

[B171] Mulcahy H. Sibley C. D. Surette M. G. Lewenza S. (2011). *Drosophila melanogaster* as an animal model for the study of *Pseudomonas aeruginosa* Biofilm Infections in vivo. *PLoS Pathog.* 7:e1002299 10.1371/journal.ppat.1002299 PMC3188550 21998591

[B172] Musher D. M. Thorner A. R. (2014). Community-acquired pneumonia. *N. Engl. J. Med.* 371 1619–1628 10.1056/NEJMra1312885 25337751

[B173] Mustafa T. Phyu S. Nilsen R. Bjune G. Jonsson R. (1999a). Increased expression of Fas ligand on *Mycobacterium tuberculosis* infected macrophages: a potential novel mechanism of immune evasion by *Mycobacterium tuberculosis*? *Inflammation* 23 507–521. 10565565 10.1023/a:1020286305950

[B174] Mustafa T. Phyu S. Nilsen R. Jonsson R. Bjune G. (1999b). A mouse model for slowly progressive primary tuberculosis. *Scand. J. Immunol.* 50 127–136 10.1046/j.1365-3083.1999.00596.x 10447916

[B175] Mustafa T. Phyu S. Nilsen R. Jonsson R. Bjune G. (2000). In situ expression of cytokines and cellular phenotypes in the lungs of mice with slowly progressive primary tuberculosis. *Scand. J. Immunol.* 51 548–556 10.1046/j.1365-3083.2000.00721.x 10849364

[B176] Nicoletti M. Iacobino A. Prosseda G. Fiscarelli E. Zarrilli R. De Carolis E. (2011). *Stenotrophomonas maltophilia* strains from cystic fibrosis patients: genomic variability and molecular characterization of some virulence determinants. *Int. J. Med. Microbiol.* 301 34–43 10.1016/j.ijmm.2010.07.003 20952251

[B177] Oh C.-T. Moon C. Choi T. H. Kim B. S. Jang J. (2013). *Mycobacterium marinum* infection in *Drosophila melanogaster* for antimycobacterial activity assessment. *J. Antimicrob. Chemother.* 68 601–609 10.1093/jac/dks425 23118147

[B178] Oksanen K. E. Halfpenny N. J. A. Sherwood E. Harjula S.-K. E. Hammarén M. M. Ahava M. J. (2013). An adult zebrafish model for preclinical tuberculosis vaccine development. *Vaccine* 31 5202–5209 10.1016/j.vaccine.2013.08.093 24055305

[B179] O’Quinn A. L. Wiegand E. M. Jeddeloh J. A. (2001). *Burkholderia pseudomallei* kills the nematode *Caenorhabditis elegans* using an endotoxin-mediated paralysis. *Cell. Microbiol.* 3 381–393 10.1046/j.1462-5822.2001.00118.x 11422081

[B180] Orme I. M. (2005a). Current progress in tuberculosis vaccine development. *Vaccine* 23 2105–2108 10.1016/j.vaccine.2005.01.062 15755579

[B181] Orme I. M. (2005b). Mouse and guinea pig models for testing new tuberculosis vaccines. *Tuberculosis (Edinb.)* 85 13–17 10.1016/j.tube.2004.08.001 15687022

[B182] Page D. M. Wittamer V. Bertrand J. Y. Lewis K. L. Pratt D. N. Delgado N. (2013). An evolutionarily conserved program of B-cell development and activation in zebrafish. *Blood* 122 e1–e11 10.1182/blood-2012-12-471029 23861249 PMC3750348

[B183] Parikka M. Hammaren M. M. Harjula S.-K. E. Halfpenny N. J. A. Oksanen K. E. Lahtinen M. J. (2012). *Mycobacterium marinum* causes a latent infection that can be reactivated by gamma irradiation in adult zebrafish. *PLoS Pathog.* 8:e1002944 10.1371/journal.ppat.1002944 PMC3459992 23028333

[B184] Park B. Iwase T. Liu G. Y. (2011). Intranasal application of *S. epidermidis* prevents colonization by Methicillin-resistant Staphylococcus aureus in mice. *PLoS ONE* 6:e25880 10.1371/journal.pone.0025880 PMC3187813 21998712

[B185] Phennicie R. T. Sullivan M. J. Singer J. T. Yoder J. A. Kim C. H. (2010). Specific resistance to *Pseudomonas aeruginosa* infection in zebrafish is mediated by the cystic fibrosis transmembrane conductance regulator. *Infect. Immun.* 78 4542–4550 10.1128/IAI.00302-10 20732993 PMC2976322

[B186] Phyu S. Mustafa T. Hofstad T. Nilsen R. Fosse R. Bjune G. (1998). A mouse model for latent tuberculosis. *Scand. J. Infect. Dis.* 30 59–68 10.1080/003655498750002321 9670361

[B187] Pier G. B. Small G. J. Warren H. B. (1990). Protection against mucoid *Pseudomonas aeruginosa* in rodent models of endobronchial infections. *Science* 249 537–540 10.1126/science.2116663 2116663

[B188] Pilátová M. Dionne M. S. (2012). *Burkholderia thailandensis* is virulent in *Drosophila melanogaster*. *PLoS ONE* 7:e49745 10.1371/journal.pone.0049745 PMC3507839 23209596

[B189] Polakowska K. Lis M. W. Helbin W. M. Dubin G. Dubin A. Niedziolka J. W. (2012). The virulence of *Staphylococcus aureus* correlates with strain genotype in a chicken embryo model but not a nematode model. *Microbes Infect.* 14 1352–1362 10.1016/j.micinf.2012.09.006 23041460

[B190] Polverino E. Torres A. Menendez R. Cillóniz C. Valles J. M. Capelastegui A. (2013). Microbial aetiology of healthcare associated pneumonia in Spain: a prospective, multicentre, case-control study. *Thorax* 68 1007–1014 10.1136/thoraxjnl-2013-203828 24130227

[B191] Pompilio A. Pomponio S. Crocetta V. Gherardi G. Verginelli F. Fiscarelli E. (2011). Phenotypic and genotypic characterization of *Stenotrophomonas maltophilia* isolates from patients with cystic fibrosis: genome diversity, biofilm formation, and virulence. *BMC Microbiol.* 11:159 10.1186/1471-2180-11-159 PMC3146419 21729271

[B192] Pozos T. C. Ramakrishnan L. Ramakrishan L. (2004). New models for the study of *Mycobacterium*-host interactions. *Curr. Opin. Immunol.* 16 499–505 10.1016/j.coi.2004.05.011 15245746

[B193] Prajsnar T. K. Cunliffe V. T. Foster S. J. Renshaw S. A. (2008). A novel vertebrate model of *Staphylococcus aureus* infection reveals phagocyte-dependent resistance of zebrafish to non-host specialized pathogens. *Cell. Microbiol.* 10 2312–2325 10.1111/j.1462-5822.2008.01213.x 18715285

[B194] Proft T. Fraser J. D. (2003). Bacterial superantigens. *Clin. Exp. Immunol.* 133 299–306 10.1046/j.1365-2249.2003.02203.x 12930353 PMC1808794

[B195] Prouty M. G. Correa N. E. Barker L. P. Jagadeeswaran P. Klose K. E. (2003). Zebrafish-*Mycobacterium marinum* model for mycobacterial pathogenesis. *FEMS Microbiol. Lett.* 225 177–182 10.1016/S0378-1097(03)00446-4 12951238

[B196] Purves J. Cockayne A. Moody P. C. E. Morrissey J. A. (2010). Comparison of the regulation, metabolic functions, and roles in virulence of the glyceraldehyde-3-phosphate dehydrogenase homologues gapA and gapB in *Staphylococcus aureus*. *Infect. Immun.* 78 5223–5232 10.1128/IAI.00762-10 20876289 PMC2981307

[B197] Rajagopalan G. Sen M. M. Singh M. Murali N. S. Nath K. A. Iijima K. (2006). Intranasal exposure to staphylococcal enterotoxin B elicits an acute systemic inflammatory response. *Shock* 25 647–656 10.1097/01.shk.0000209565.92445.7d 16721274

[B198] Ramachandran S. Ruef B. Pich C. Sprague J. (2010). Exploring zebrafish genomic, functional and phenotypic data using ZFIN. *Curr. Protoc. Bioinformatics Chap.* 1 Unit 1.18. 10.1002/0471250953.bi0118s31 PMC4562798 20836073

[B199] Ramakrishnan L. (2013). Looking within the zebrafish to understand the tuberculous granuloma. *Adv. Exp. Med. Biol.* 783 251–266 10.1007/978-1-4614-6111-1_13 23468113

[B200] Ramarao N. Nielsen-Leroux C. Lereclus D. (2012). The Insect *Galleria mellonella* as a powerful infection model to investigate bacterial pathogenesis. *J. Vis. Exp.* 70:e4392 10.3791/4392 PMC3567165 23271509

[B201] Ratner A. J. Bryan R. Weber A. Nguyen S. Barnes D. Pitt A. (2001). Cystic fibrosis pathogens activate Ca^2+^-dependent mitogen-activated protein kinase signaling pathways in airway epithelial cells. *J. Biol. Chem.* 276 19267–19275 10.1074/jbc.M007703200 11278360

[B202] Rauta P. R. Nayak B. Das S. (2012). Immune system and immune responses in fish and their role in comparative immunity study: a model for higher organisms. *Immunol. Lett.* 148 23–33 10.1016/j.imlet.2012.08.003 22902399

[B203] Rayner C. F. Jackson A. D. Rutman A. Dewar A. Mitchell T. J. Andrew P. W. (1995). Interaction of pneumolysin-sufficient and -deficient isogenic variants of *Streptococcus pneumoniae* with human respiratory mucosa. *Infect. Immun.* 63 442–447. 7822008 10.1128/iai.63.2.442-447.1995 PMC173015

[B204] Redd M. J. Kelly G. Dunn G. Way M. Martin P. (2006). Imaging macrophage chemotaxis in vivo: studies of microtubule function in zebrafish wound inflammation. *Cell Motil. Cytoskeleton* 63 415–422 10.1002/cm.20133 16671106

[B205] Renshaw S. A. Loynes C. A. Elworthy S. Ingham P. W. Whyte M. K. B. (2007). Modeling inflammation in the zebrafish: how a fish can help us understand lung disease. *Exp. Lung Res.* 33 549–554 10.1080/01902140701756778 18075830

[B206] Rodríguez-Rojas A. Mena A. Martín S. Borrell N. Oliver A. Blázquez J. (2009). Inactivation of the hmgA gene of *Pseudomonas aeruginosa* leads to pyomelanin hyperproduction, stress resistance and increased persistence in chronic lung infection. *Microbiology* 155 1050–1057 10.1099/mic.0.024745-0 19332807

[B207] Rombough P. (2007). The functional ontogeny of the teleost gill: which comes first, gas or ion exchange? *Comp. Biochem. Physiol. A Mol. Integr. Physiol. 148*, 732–742 10.1016/j.cbpa.2007.03.007 17451987

[B208] Rounioja S. Saralahti A. Rantala L. Parikka M. Henriques-Normark B. Silvennoinen O. (2012). Defense of zebrafish embryos against *Streptococcus pneumoniae* infection is dependent on the phagocytic activity of leukocytes. *Dev. Comp. Immunol.* 36 342–348 10.1016/j.dci.2011.05.008 21658407

[B209] Russell W. M. S. (1995). The development of the three Rs concept. *Altern. Lab. Anim.* 23 298–304. 11656565

[B210] Russell W. Burch R. (1959). *The Principles of Humane Experimental Technique.* London: Methuen.

[B211] Ruyra A. Cano-Sarabia M. García-Valtanen P. Yero D. Gibert I. Mackenzie S. A. (2014). Targeting and stimulation of the zebrafish (*Danio rerio*) innate immune system with LPS/dsRNA-loaded nanoliposomes. *Vaccine* 32 3955–3962 10.1016/j.vaccine.2014.05.010 24837767

[B212] Saini D. Hopkins G. W. Seay S. A. Chen C.-J. Perley C. C. Click E. M. (2012). Ultra-low dose of *Mycobacterium tuberculosis* aerosol creates partial infection in mice. *Tuberculosis (Edinb.)* 92 160–165 10.1016/j.tube.2011.11.007 22197183 PMC3288716

[B213] Sapru K. Stotland P. K. Stevenson M. M. (1999). Quantitative and qualitative differences in bronchoalveolar inflammatory cells in *Pseudomonas aeruginosa*-resistant and -susceptible mice. *Clin. Exp. Immunol.* 115 103–109 10.1046/j.1365-2249.1999.00762.x 9933427 PMC1905184

[B214] Saralahti A. Piippo H. Parikka M. Henriques-Normark B. Rämet M. Rounioja S. (2014). Adult zebrafish model for pneumococcal pathogenesis. *Dev. Comp. Immunol.* 42 345–353 10.1016/j.dci.2013.09.009 24076065

[B215] Sawai T. Tomono K. Yanagihara K. Yamamoto Y. Kaku M. Hirakata Y. (1997). Role of coagulase in a murine model of hematogenous pulmonary infection induced by intravenous injection of *Staphylococcus aureus* enmeshed in agar beads. *Infect. Immun.* 65 466–471. 9009298 10.1128/iai.65.2.466-471.1997 PMC174618

[B216] Schulenburg H. Ewbank J. J. (2007). The genetics of pathogen avoidance in *Caenorhabditis elegans*. *Mol. Microbiol.* 66 563–570 10.1111/j.1365-2958.2007.05946.x 17877707

[B217] Seidenfeld J. J. Mullins R. C. III Fowler S. R. Johanson W. G. Jr. (1986). Bacterial infection and acute lung injury in hamsters. *Am. Rev. Respir. Dis.* 134 22–26. 3729158 10.1164/arrd.1986.134.1.22

[B218] Shi C. Shi J. Xu Z. (2011). A review of murine models of latent tuberculosis infection. *Scand. J. Infect. Dis.* 43 848–856 10.3109/00365548.2011.603745 21892898

[B219] Shivers R. P. Pagano D. J. Kooistra T. Richardson C. E. Reddy K. C. Whitney J. K. (2010). Phosphorylation of the conserved transcription factor ATF-7 by PMK-1 p38 MAPK regulates innate immunity in *Caenorhabditis elegans*. *PLoS Genet.* 6:e1000892 10.1371/journal.pgen.1000892 PMC2848548 20369020

[B220] Sifri C. D. Baresch-Bernal A. Calderwood S. B. von Eiff C. (2006). Virulence of *Staphylococcus aureus* small colony variants in the *Caenorhabditis elegans* infection model. *Infect. Immun.* 74 1091–1096 10.1128/IAI.74.2.1091-1096.2006 16428756 PMC1360298

[B221] Sifri C. D. Begun J. Ausubel F. M. (2005). The worm has turned – microbial virulence modeled in *Caenorhabditis elegans*. *Trends Microbiol.* 13 119–127 10.1016/j.tim.2005.01.003 15737730

[B222] Sifri C. D. Begun J. Ausubel F. M. Calderwood S. B. (2003). *Caenorhabditis elegans* as a model host for *Staphylococcus aureus* pathogenesis. *Infect. Immun.* 71 2208–2217 10.1128/IAI.71.4.2208-2217.2003 12654843 PMC152095

[B223] Smith M. P. Laws T. R. Atkins T. P. Oyston P. C. F. de Pomerai D. I. Titball R. W. (2002). A liquid-based method for the assessment of bacterial pathogenicity using the nematode *Caenorhabditis elegans*. *FEMS Microbiol. Lett.* 210 181–185 10.1111/j.1574-6968.2002.tb11178.x 12044672

[B224] Sprynski N. Valade E. Neulat-Ripoll F. (2014). *Galleria mellonella* as an infection model for select agents. *Methods Mol. Biol.* 1197 3–9 10.1007/978-1-4939-1261-2_1 25172272

[B225] Srivastava A. Casey H. Johnson N. Levy O. Malley R. (2007). Recombinant bactericidal/permeability-increasing protein rBPI21 protects against pneumococcal disease. *Infect. Immun.* 75 342–349 10.1128/IAI.01089-06 17101667 PMC1828387

[B226] Staczek J. Gilleland H. E. Jr. Gilleland L. B. Harty R. N. García-Sastre A. Engelhardt O. G. (1998). A chimeric influenza virus expressing an epitope of outer membrane protein F of *Pseudomonas aeruginosa* affords protection against challenge with *P.* aeruginosa in a murine model of chronic pulmonary infection. *Infect. Immun.* 66 3990–3994. 9673294 10.1128/iai.66.8.3990-3994.1998 PMC108472

[B227] Starke J. R. Edwards M. S. Langston C. Baker C. J. (1987). A mouse model of chronic pulmonary infection with *Pseudomonas aeruginosa* and *Pseudomonas cepacia*. *Pediatr. Res.* 22 698–702 10.1203/00006450-198712000-00017 3431954

[B228] Stevens C. W. (1992). Alternatives to the use of mammals for pain research. *Life Sci.* 50 901–912 10.1016/0024-3205(92)90167-N 1548975

[B229] Stevenson M. M. Kondratieva T. K. Apt A. S. Tam M. F. Skamene E. (1995). In vitro and in vivo T cell responses in mice during bronchopulmonary infection with mucoid *Pseudomonas aeruginosa*. *Clin. Exp. Immunol.* 99 98–105 10.1111/j.1365-2249.1995.tb03478.x 7813116 PMC1534146

[B230] Strähle U. Scholz S. Geisler R. Greiner P. Hollert H. Rastegar S. (2012). Zebrafish embryos as an alternative to animal experiments–a commentary on the definition of the onset of protected life stages in animal welfare regulations. *Reprod. Toxicol.* 33 128–132 10.1016/j.reprotox.2011.06.121 21726626

[B231] Stundick M. V. Albrecht M. T. Houchens C. R. Smith A. P. Dreier T. M. Larsen J. C. (2013). Animal models for *Francisella tularensis* and *Burkholderia* species: scientific and regulatory gaps toward approval of antibiotics under the FDA animal rule. *Vet. Pathol.* 50 877–892 10.1177/0300985813486812 23628693

[B232] Sulston J. Hodgkin J. (1988). “Methods,” in *The Nematode Caenorhabditis elegans*, ed. Wood W. B. (New York, NY: Cold Spring Harbor Laboratory Press), 587–606.

[B233] Sunyer J. O. Boshra H. Li J. (2005). Evolution of anaphylatoxins, their diversity and novel roles in innate immunity: insights from the study of fish complement. *Vet. Immunol. Immunopathol.* 108 77–89 10.1016/j.vetimm.2005.07.009 16112742

[B234] Swaim L. E. Connolly L. E. Volkman H. E. Humbert O. Born D. E. Ramakrishnan L. (2006). *Mycobacterium marinum* infection of adult zebrafish causes caseating granulomatous tuberculosis and is moderated by adaptive immunity. *Infect. Immun.* 74 6108–6117 10.1128/IAI.00887-06 17057088 PMC1695491

[B235] Takaki K. Davis J. M. Winglee K. Ramakrishnan L. (2013). Evaluation of the pathogenesis and treatment of *Mycobacterium marinum* infection in zebrafish. *Nat. Protoc.* 8 1114–1124 10.1038/nprot.2013.068 23680983 PMC3919459

[B236] Tam M. Snipes G. J. Stevenson M. M. (1999). Characterization of chronic bronchopulmonary *Pseudomonas aeruginosa* infection in resistant and susceptible inbred mouse strains. *Am. J. Respir. Cell Mol. Biol.* 20 710–719 10.1165/ajrcmb.20.4.3223 10101003

[B237] Tan M.-W. (2002). Identification of host and pathogen factors involved in virulence using *Caenorhabditis elegans*. *Methods Enzymol.* 358 13–28 10.1016/S0076-6879(02)58078-2 12474376

[B238] Tan M. W. Ausubel F. M. (2000). *Caenorhabditis elegans*: a model genetic host to study *Pseudomonas aeruginosa* pathogenesis. *Curr. Opin. Microbiol.* 3 29–34 10.1016/S1369-5274(99)00047-8 10679415

[B239] Tan M. W. Mahajan-Miklos S. Ausubel F. M. (1999a). Killing of *Caenorhabditis elegans* by *Pseudomonas aeruginosa* used to model mammalian bacterial pathogenesis. *Proc. Natl. Acad. Sci. U.S.A.* 96 715–720 10.1073/pnas.96.2.715 9892699 PMC15202

[B240] Tan M. W. Rahme L. G. Sternberg J. A. Tompkins R. G. Ausubel F. M. (1999b). *Pseudomonas aeruginosa* killing of *Caenorhabditis elegans* used to identify *P. aeruginosa* virulence factors. Proc. Natl. Acad. Sci. U.S.A. 96 2408–2413 10.1073/pnas.96.5.2408 10051655 PMC26797

[B241] Teles R. Wang C. Y. Stashenko P. (1997). Increased susceptibility of RAG-2 SCID mice to dissemination of endodontic infections. *Infect. Immun.* 65 3781–3787. 9284152 10.1128/iai.65.9.3781-3787.1997 PMC175539

[B242] Terashima T. Kanazawa M. Sayama K. Urano T. Sakamaki F. Nakamura H. (1995). Neutrophil-induced lung protection and injury are dependent on the amount of *Pseudomonas aeruginosa* administered via airways in guinea pigs. *Am. J. Respir. Crit. Care Med.* 152 2150–2156 10.1164/ajrccm.152.6.8520789 8520789

[B243] Thomas R. Hamat R. A. Neela V. (2013). *Stenotrophomonas maltophilia*: pathogenesis model using *Caenorhabditis elegans*. *J. Med. Microbiol.* 62 1777–1779 10.1099/jmm.0.063230-0 23988629

[B244] Tobin D. M. Ramakrishnan L. (2008). Comparative pathogenesis of *Mycobacterium marinum* and *Mycobacterium tuberculosis*. *Cell. Microbiol.* 10 1027–1039 10.1111/j.1462-5822.2008.01133.x 18298637

[B245] TranVan Nhieu G. Clair C. Grompone G. Sansonetti P. (2004). Calcium signalling during cell interactions with bacterial pathogens. *Biol. Cell* 96 93–101 10.1016/j.biolcel.2003.10.006 15093131

[B246] van der Sar A. M. Abdallah A. M. Sparrius M. Reinders E. Vandenbroucke-Grauls C. M. J. E. Bitter W. (2004a). *Mycobacterium marinum* strains can be divided into two distinct types based on genetic diversity and virulence. *Infect. Immun.* 72 6306–6312 10.1128/IAI.72.11.6306-6312.2004 15501758 PMC523024

[B247] van der Sar A. M. Appelmelk B. J. Vandenbroucke-Grauls C. M. J. E. Bitter W. (2004b). A star with stripes: zebrafish as an infection model. *Trends Microbiol.* 12 451–457 10.1016/j.tim.2004.08.001 15381194

[B248] van Heeckeren A. M. Schluchter M. D. (2002). Murine models of chronic *Pseudomonas aeruginosa* lung infection. *Lab. Anim.* 36 291–312 10.1258/002367702320162405 12144741

[B249] Vilaplana C. Cardona P.-J. (2014). The lack of a big picture in tuberculosis: the clinical point of view, the problems of experimental modeling and immunomodulation. The factors we should consider when designing novel treatment strategies. *Front. Microbiol.* 5:55 10.3389/fmicb.2014.00055 PMC3924323 24592258

[B250] Volkman H. E. Clay H. Beery D. Chang J. C. W. Sherman D. R. Ramakrishnan L. (2004). Tuberculous granuloma formation is enhanced by a mycobacterium virulence determinant. *PLoS Biol.* 2:e367 10.1371/journal.pbio.0020367 PMC524251 15510227

[B251] Volkman H. E. Pozos T. C. Zheng J. Davis J. M. Rawls J. F. Ramakrishnan L. (2010). Tuberculous granuloma induction via interaction of a bacterial secreted protein with host epithelium. *Science* 327 466–469 10.1126/science.1179663 20007864 PMC3125975

[B252] Wand M. E. Müller C. M. Titball R. W. Michell S. L. (2011). Macrophage and *Galleria mellonella* infection models reflect the virulence of naturally occurring isolates of *B. pseudomallei, B. thailandensis and B. oklahomensis. BMC Microbiol.* 11:11 10.1186/1471-2180-11-11 PMC3025829 21241461

[B253] Warawa J. M. (2010). Evaluation of surrogate animal models of melioidosis. *Front. Microbiol.* 1:141 10.3389/fmicb.2010.00141 PMC3109346 21772830

[B254] Waters V. J. Gómez M. I. Soong G. Amin S. Ernst R. K. Prince A. (2007). Immunostimulatory properties of the emerging pathogen *Stenotrophomonas maltophilia*. *Infect. Immun.* 75 1698–1703 10.1128/IAI.01469-06 17220304 PMC1865680

[B255] Watkins-Chow D. E. Pavan W. J. (2008). Genomic copy number and expression variation within the C57BL/6J inbred mouse strain. *Genome Res.* 18 60–66 10.1101/gr.6927808 18032724 PMC2134784

[B256] Wilmott R. W. Kitzmiller J. A. Szabó C. Southan G. J. Salzman A. L. (2000). Mercaptoethylguanidine inhibits the inflammatory response in a murine model of chronic infection with *Pseudomonas aeruginosa*. *J. Pharmacol. Exp. Ther.* 292 88–95. 10604934

[B257] Woodhead M. (2013). Pneumonia classification and healthcare-associated pneumonia: a new avenue or just a cul-de-sac? *Thorax* 68 985–986 10.1136/thoraxjnl-2013-204060 24002056

[B258] Wu K. Conly J. McClure J.-A. Elsayed S. Louie T. Zhang K. (2010). *Caenorhabditis elegans* as a host model for community-associated methicillin-resistant *Staphylococcus aureus*. *Clin. Microbiol. Infect.* 16 245–254 10.1111/j.1469-0691.2009.02765.x 19456837

[B259] Wu K. Conly J. Surette M. Sibley C. Elsayed S. Zhang K. (2012). Assessment of virulence diversity of methicillin-resistant *Staphylococcus aureus* strains with a *Drosophila melanogaster* infection model. *BMC Microbiol.* 12:274 10.1186/1471-2180-12-274 PMC3539928 23176146

[B260] Wu K. Zhang K. McClure J. Zhang J. Schrenzel J. Francois P. (2013). A correlative analysis of epidemiologic and molecular characteristics of methicillin-resistant *Staphylococcus aureus* clones from diverse geographic locations with virulence measured by a *Caenorhabditis elegans* host model. *Eur. J. Clin. Microbiol. Infect. Dis.* 32 33–42 10.1007/s10096-012-1711-x 22898726 PMC3545200

[B261] Xu J. Du L. Wen Z. (2012). Myelopoiesis during zebrafish early development. *J. Genet. Genomics* 39 435–442 10.1016/j.jgg.2012.06.005 23021543

[B262] Young D. (2009). Animal models of tuberculosis. *Eur. J. Immunol.* 39 2011–2014 10.1002/eji.200939542 19672894

[B263] Yuen G. J. Ausubel F. M. (2014). *Enterococcus* infection biology: lessons from invertebrate host models. *J. Microbiol.* 52 200–210 10.1007/s12275-014-4011-6 24585051 PMC4556283

[B264] Zak O. O’Reilly T. (1993). Animal infection models and ethics – the perfect infection model. *J. Antimicrob. Chemother.* 31 193–205 10.1093/jac/31.suppl_D.193 11660028

[B265] Zhang T. Li S.-Y. Williams K. N. Andries K. Nuermberger E. L. (2011). Short-course chemotherapy with TMC207 and rifapentine in a murine model of latent tuberculosis infection. *Am. J. Respir. Crit. Care Med.* 184 732–737 10.1164/rccm.201103-0397OC 21659613 PMC3208599

